# Supplementary material for: Engaging Artificial Intelligence (AI)-based chatbots in digital health: A systematic review
Source: PLOS Digit Health. 2026 Feb 12;5(2):e0001201. doi: 10.1371/journal.pdig.0001201 (PMC12900317; doi:10.1371/journal.pdig.0001201)
Supplement: S2 Appendix — (DOCX) [file pdig.0001201.s002.docx]

The supplementary material: the list of 348 articles from screening

1. Abavisani, M., Khoshrou, A., Karbas Foroushan, S., & Sahebkar, A. (2024). Chatting with artificial intelligence to combat antibiotic resistance: Opportunities and challenges. *Current Research in Biotechnology*, *7*, 100197. https://doi.org/10.1016/j.crbiot.2024.100197
2. Abbas, T., Gadiraju, U., Khan, V.-J., & Markopoulos, P. (2022). Understanding User Perceptions of Response Delays in Crowd-Powered Conversational Systems. In *Proc. ACM Hum.-Comput. Interact.* (Vol. 6, Issue CSCW2, p. Article 345). Association for Computing Machinery.
3. Abstracts from the 2023 Annual Meeting of the Society of General Internal Medicine. (2023). *Journal of General Internal Medicine*, *38*(S2), 81–799. https://doi.org/10.1007/s11606-023-08226-z
4. Afrizal, S. H., Hakiem, N., Erna Permanasari, A., Syaifullah Albab, H., Yoki Sanjaya, G., & Lazuardi, L. (2022). A User-Centered Design of Natural Language Processing for Maternal Monitoring Chatbot System. *2022 International Conference on Informatics, Multimedia, Cyber and Information System (ICIMCIS)*, 244–248. https://doi.org/10.1109/ICIMCIS56303.2022.10017517
5. Aggarwal, A., Tam, C. C., Wu, D., Li, X., & Qiao, S. (2022). *Artificial Intelligence (AI)-based Chatbots in Promoting Health Behavioral Changes: A Systematic Review*. https://doi.org/10.1101/2022.07.05.22277263
6. Aggarwal, A., Tam, C. C., Wu, D., Li, X., & Qiao, S. (2023). Artificial Intelligence–Based Chatbots for Promoting Health Behavioral Changes: Systematic Review. *Journal of Medical Internet Research*, *25*, e40789. https://doi.org/10.2196/40789
7. Ahmad, A., Premanandan, S., Cajander, Å., Langegård, U., Uereten, E., & Tiblom Ehrsson, Y. (2024). A Qualitative Study with Informal Caregivers and Healthcare Professionals for Individuals with Head and Neck Cancer on the Usage of AI Chatbots. In J. Mantas, A. Hasman, G. Demiris, K. Saranto, M. Marschollek, T. N. Arvanitis, I. Ognjanović, A. Benis, P. Gallos, E. Zoulias, & E. Andrikopoulou (Eds.), *Studies in Health Technology and Informatics*. IOS Press. https://doi.org/10.3233/SHTI240522
8. Ahmadi, S., & Fox, E. A. (2024). AI Chatbot for Generating Episodic Future Thinking (EFT) Cue Texts for Health. *2024 IEEE First International Conference on Artificial Intelligence for Medicine, Health and Care (AIMHC)*, 101–108. https://doi.org/10.1109/AIMHC59811.2024.00027
9. Ahmed, F. R., Al-Yateem, N., Rushdan, E., Saifan, A. R., Rahman, S., Mottershead, R., Hijazi, H., Subu, M. A., Bani-Issa, W., Dias, J. M., & Aburuz, M. E. (2024). Health Sciences students and educators experience with using ChatGPT. *2024 IEEE 48th Annual Computers, Software, and Applications Conference (COMPSAC)*, 1926–1928. https://doi.org/10.1109/COMPSAC61105.2024.00305
10. Akhtar, M. M., Karunanayake, I., Sharma, B., Masood, R., Ikram, M., & Kanhere, S. S. (2023). Towards Automatic Annotation and Detection of Fake News. *2023 IEEE 48th Conference on Local Computer Networks (LCN)*, 1–9. https://doi.org/10.1109/LCN58197.2023.10223359
11. Al Naabi, Y., Ibrahim, N., & Dhillon, J. S. (2024). Designing sustainable mobile weight management applications: Information technology (IT) experts perspectives. *mHealth*, *10*, 25–25. https://doi.org/10.21037/mhealth-24-4
12. Alanezi, F. (2024). Factors influencing patients’ engagement with ChatGPT for accessing health-related information. *Critical Public Health*, *34*(1), 1–20. https://doi.org/10.1080/09581596.2024.2348164
13. Alberts, L., Lyngs, U., & Kleek, M. V. (2024). Computers as Bad Social Actors: Dark Patterns and Anti-Patterns in Interfaces that Act Socially. In *Proc. ACM Hum.-Comput. Interact.* (Vol. 8, Issue CSCW1, p. Article 202). Association for Computing Machinery.
14. Albino De Queiroz, D., Silva Passarello, R., Veloso De Moura Fé, V., Rossini, A., Folchini Da Silveira, E., Aparecida Isquierdo Fonseca De Queiroz, E., & André Da Costa, C. (2023). A wearable chatbot-based model for monitoring colorectal cancer patients in the active phase of treatment. *Healthcare Analytics*, *4*, 100257. https://doi.org/10.1016/j.health.2023.100257
15. Alikhanov, J., Zhang, P., Noh, Y., & Kim, H. (2024). Design of Contextual Filtered Features for Better Smartphone-User Receptivity Prediction. *IEEE Internet of Things Journal*, *11*(7), 11707–11722. https://doi.org/10.1109/JIOT.2023.3331715
16. Ambrosio, M. D. G., Lachman, J. M., Zinzer, P., Gwebu, H., Vyas, S., Vallance, I., Calderon, F., Gardner, F., Markle, L., Stern, D., Facciola, C., Schley, A., Danisa, N., Brukwe, K., & Melendez-Torres, G. (2024). A Factorial Randomized Controlled Trial to Optimize User Engagement With a Chatbot-Led Parenting Intervention: Protocol for the ParentText Optimisation Trial. *JMIR Research Protocols*, *13*, e52145. https://doi.org/10.2196/52145
17. Amin, M. M., Mao, R., Cambria, E., & Schuller, B. W. (2024). A Wide Evaluation of ChatGPT on Affective Computing Tasks. *IEEE Transactions on Affective Computing*, 1–9. https://doi.org/10.1109/TAFFC.2024.3419593
18. Andreadis, K., Rodriguez, D. V., Zakreuskaya, A., Chen, J., Gonzalez, J., & Mann, D. (2024). Bridging Gaps with Generative AI: Enhancing Hypertension Monitoring Through Patient and Provider Insights. In J. Mantas, A. Hasman, G. Demiris, K. Saranto, M. Marschollek, T. N. Arvanitis, I. Ognjanović, A. Benis, P. Gallos, E. Zoulias, & E. Andrikopoulou (Eds.), *Studies in Health Technology and Informatics*. IOS Press. https://doi.org/10.3233/SHTI240565
19. Andrews, N. E., Ireland, D., Vijayakumar, P., Burvill, L., Hay, E., Westerman, D., Rose, T., Schlumpf, M., Strong, J., & Claus, A. (2023). Acceptability of a Pain History Assessment and Education Chatbot (Dolores) Across Age Groups in Populations With Chronic Pain: Development and Pilot Testing. *JMIR Formative Research*, *7*, e47267. https://doi.org/10.2196/47267
20. Aniza, A. N., Afiqah, M. S. N., Ikhwan, M. K. M., Rozilawati, A., Azmie, Y. N., Safwan, A. F. M., Adilah, S. N., Noraini, A. W., Izzuan, H. S., & Liyana, S. (2023). REVOLUTIONIZING RADIOTHERAPY COMMUNICATION SKILLS TRAINING: AN INNOVATIVE CHATBOT-BASED PROTOTYPE APPROACH (SCIMORT). *Journal of Medical Imaging and Radiation Sciences*, *54*(3), S14–S15. https://doi.org/10.1016/j.jmir.2023.06.052
21. Anmella, G., Sanabra, M., Primé-Tous, M., Segú, X., Cavero, M., Morilla, I., Grande, I., Ruiz, V., Mas, A., Martín-Villalba, I., Caballo, A., Esteva, J.-P., Rodríguez-Rey, A., Piazza, F., Valdesoiro, F. J., Rodriguez-Torrella, C., Espinosa, M., Virgili, G., Sorroche, C., … Hidalgo-Mazzei, D. (2023). Vickybot, a Chatbot for Anxiety-Depressive Symptoms and Work-Related Burnout in Primary Care and Health Care Professionals: Development, Feasibility, and Potential Effectiveness Studies. *Journal of Medical Internet Research*, *25*, e43293. https://doi.org/10.2196/43293
22. Anmella, G., Sanabra, M., Primé-tous, M., Segú, X., Cavero, M., Navinés, R., Mas, A., Olivé, V., Pujol, L., Quesada, S., Pio, C., Villegas, M., Grande, I., Morilla, I., Martínez-Aran, A., Ruiz, V., Vieta, E., & Hidalgo-Mazzei, D. (2023). Vickybot, a chatbot for anxiety-depressive symptoms and work-related burnout. *European Psychiatry*, *66*(S1), S109–S110. https://doi.org/10.1192/j.eurpsy.2023.301
23. Antonini, A., Adamou, A., Suárez-Figueroa, M. C., & Benatti, F. (2023). Experiential Observations: An Ontology Pattern-Based Study on Capturing the Potential Content within Evidences of Experiences. In *J. Comput. Cult. Herit.* (Vol. 16, Issue 3, p. Article 58). Association for Computing Machinery.
24. Antony, V. N., & Huang, C.-M. (2024). ID.8: Co-Creating Visual Stories with Generative AI. In *ACM Trans. Interact. Intell. Syst.* (Vol. 14, Issue 3, p. Article 20). Association for Computing Machinery.
25. Anuyah, O., Conrado, A.-M., Carlson, C., Gilbride, H., & Metoyer, R. (2023). Exploring the Barriers and Potential Opportunities of Technology Integration in Community-Based Social Service Organizations. In *ACM J. Comput. Sustain. Soc.* (Vol. 1, Issue 1, p. Article 7). Association for Computing Machinery.
26. Arun, A., K V, A. B., Rajesh, G., H, P., & Bhadran, B. (2024). Transforming Healthcare: Unified Medical Identification and AI-Enabled Treatment Advancements. *2024 Second International Conference on Inventive Computing and Informatics (ICICI)*, 378–382. https://doi.org/10.1109/ICICI62254.2024.00068
27. Ashton, L. M., Adam, M. T., Whatnall, M., Rollo, M. E., Burrows, T. L., Hansen, V., & Collins, C. E. (2023). Exploring the design and utility of an integrated web-based chatbot for young adults to support healthy eating: A qualitative study. *International Journal of Behavioral Nutrition and Physical Activity*, *20*(1), 119. https://doi.org/10.1186/s12966-023-01511-4
28. ATTD 2023 Abstract Author Index. (2023). *Diabetes Technology & Therapeutics*, *25*(S2), A-270-A-284. https://doi.org/10.1089/dia.2023.2526.abstracts.index
29. Awori, K., Allela, M. A., Nyairo, S., Maina, S. C., & O’Neill, J. (2022). “It’s only when somebody says a tool worked for them that I believe it will work for me”: Socio-tecture as a lens for Digital Transformation. In *Proc. ACM Hum.-Comput. Interact.* (Vol. 6, Issue CSCW2, p. Article 483). Association for Computing Machinery.
30. Ayobi, A., Eardley, R., Soubutts, E., Gooberman-Hill, R., Craddock, I., & O’Kane, A. A. (2022). Digital Mental Health and Social Connectedness: Experiences of Women from Refugee Backgrounds. In *Proc. ACM Hum.-Comput. Interact.* (Vol. 6, Issue CSCW2, p. Article 507). Association for Computing Machinery.
31. Azhar, M. Q., & Haynes, A. (2022). A Pilot Study of a Virtual Informal Experiential Learning Activity during COVID-19. *J. Comput. Sci. Coll.*, *38*(5), 117–126.
32. Babu, A., & Boddu, S. B. (2024). BERT-Based Medical Chatbot: Enhancing Healthcare Communication through Natural Language Understanding. *Exploratory Research in Clinical and Social Pharmacy*, *13*, 100419. https://doi.org/10.1016/j.rcsop.2024.100419
33. Badshah, A., Ghani, A., Daud, A., Jalal, A., Bilal, M., & Crowcroft, J. (2023). Towards Smart Education through Internet of Things: A Survey. In *ACM Comput. Surv.* (Vol. 56, Issue 2, p. Article 26). Association for Computing Machinery.
34. Balaji, D., He, L., Giani, S., Bosse, T., Wiers, R., & De Bruijn, G.-J. (2022). Effectiveness and acceptability of conversational agents for sexual health promotion: A systematic review and meta-analysis. *Sexual Health*, *19*(5), 391–405. https://doi.org/10.1071/SH22016
35. Beinema, T., Op Den Akker, H., Hurmuz, M., Jansen-Kosterink, S., & Hermens, H. (2022). Automatic topic selection for long-term interaction with embodied conversational agents in health coaching: A micro-randomized trial. *Internet Interventions*, *27*, 100502. https://doi.org/10.1016/j.invent.2022.100502
36. Bendotti, H., Lawler, S., Chan, G. C. K., Gartner, C., Ireland, D., & Marshall, H. M. (2023). Conversational artificial intelligence interventions to support smoking cessation: A systematic review and meta-analysis. *DIGITAL HEALTH*, *9*, 20552076231211634. https://doi.org/10.1177/20552076231211634
37. Benítez, T. M., Xu, Y., Boudreau, J. D., Kow, A. W. C., Bello, F., Phuoc, L. V., Wang, X., Sun, X., Leung, G. K.-K., Lan, Y., Wang, Y., Cheng, D., Tham, Y.-C., Wong, T. Y., & Chung, K. C. (2024). Harnessing the potential of large language models in medical education: Promise and pitfalls. *Journal of the American Medical Informatics Association*, ocad252. https://doi.org/10.1093/jamia/ocad252
38. Bhattacharjee, A., Pang, J., Liu, A., Mariakakis, A., & Williams, J. J. (2023). Design Implications for One-Way Text Messaging Services that Support Psychological Wellbeing. In *ACM Trans. Comput.-Hum. Interact.* (Vol. 30, Issue 3, p. Article 34). Association for Computing Machinery.
39. Bhattacharjee, A., Williams, J. J., Chou, K., Tomlinson, J., Meyerhoff, J., Mariakakis, A., & Kornfield, R. (2022). “I Kind of Bounce off It”:Translating Mental Health Principles into Real Life Through Story-Based Text Messages. In *Proc. ACM Hum.-Comput. Interact.* (Vol. 6, Issue CSCW2, p. Article 398). Association for Computing Machinery.
40. Bjerregaard, A. A., Zoughbie, D. E., Hansen, J. V., Granström, C., Strøm, M., Halldórsson, Þ. I., Meder, I. K., Willett, W. C., Ding, E. L., & Olsen, S. F. (2024). An SMS chatbot digital educational program to increase healthy eating behaviors in adolescence: A multifactorial randomized controlled trial among 7,890 participants in the Danish National Birth Cohort. *PLOS Medicine*, *21*(6), e1004383. https://doi.org/10.1371/journal.pmed.1004383
41. Blanchard, M., Koller, C. N., Azevedo, P. M., Prétat, T., & Hügle, T. (2024). Development of a Management App for Postviral Fibromyalgia-Like Symptoms: Patient Preference-Guided Approach. *JMIR Formative Research*, *8*, e50832. https://doi.org/10.2196/50832
42. Bortone, G., Caro, G., Ala, L., Gargano, L., & Rossi, A. (2024). A New Method for the Follow-Up of Patients with Alopecia Areata. *Journal of Clinical Medicine*, *13*(13), 3901. https://doi.org/10.3390/jcm13133901
43. Brachman, M., Ashktorab, Z., Desmond, M., Duesterwald, E., Dugan, C., Joshi, N. N., Pan, Q., & Sharma, A. (2022). Reliance and Automation for Human-AI Collaborative Data Labeling Conflict Resolution. In *Proc. ACM Hum.-Comput. Interact.* (Vol. 6, Issue CSCW2, p. Article 321). Association for Computing Machinery.
44. Bragazzi, N. L., Crapanzano, A., Converti, M., Zerbetto, R., & Khamisy-Farah, R. (2023). The Impact of Generative Conversational Artificial Intelligence on the Lesbian, Gay, Bisexual, Transgender, and Queer Community: Scoping Review. *Journal of Medical Internet Research*, *25*, e52091. https://doi.org/10.2196/52091
45. Bricker, J. B., Sullivan, B., Mull, K., Santiago-Torres, M., & Lavista Ferres, J. M. (2024). Conversational Chatbot for Cigarette Smoking Cessation: Results From the 11-Step User-Centered Design Development Process and Randomized Controlled Trial. *JMIR mHealth and uHealth*, *12*, e57318. https://doi.org/10.2196/57318
46. Briganti, G. (2024). How ChatGPT works: A mini review. *European Archives of Oto-Rhino-Laryngology*, *281*(3), 1565–1569. https://doi.org/10.1007/s00405-023-08337-7
47. Brinsley, J., Singh, B., & Maher, C. A. (2023). A Digital Lifestyle Program for Psychological Distress, Wellbeing and Return-to-Work: A Proof-of-Concept Study. *Archives of Physical Medicine and Rehabilitation*, *104*(11), 1903–1912. https://doi.org/10.1016/j.apmr.2023.04.023
48. Bui, T. A., Pohl, M., Rosenfelt, C., Ogourtsova, T., Yousef, M., Whitlock, K., Majnemer, A., Nicholas, D., Demmans Epp, C., Zaiane, O., & Bolduc, F. V. (2022). Identifying Potential Gamification Elements for A New Chatbot for Families With Neurodevelopmental Disorders: User-Centered Design Approach. *JMIR Human Factors*, *9*(3), e31991. https://doi.org/10.2196/31991
49. Calvo, R. A., Peters, D., Moradbakhti, L., Cook, D., Rizos, G., Schuller, B., Kallis, C., Wong, E., & Quint, J. (2023). Assessing the Feasibility of a Text-Based Conversational Agent for Asthma Support: Protocol for a Mixed Methods Observational Study. *JMIR Research Protocols*, *12*, e42965. https://doi.org/10.2196/42965
50. Canha, D., Bour, C., Barraud, S., Aguayo, G., & Fagherazzi, G. (2024). The transformative role of artificial intelligence in diabetes care and research. *Diabetes & Metabolism*, *50*(5), 101565. https://doi.org/10.1016/j.diabet.2024.101565
51. Cannanure, V. K., Ngoon, T., Wolf, S., Jasińska, K., Brown, T. X., & Ogan, A. (2024). Understanding the Longitudinal Impact of a Chatbot to Facilitate a Virtual Community of Practice for Teachers in Rural Côte d’Ivoire. In *ACM J. Comput. Sustain. Soc.* (Vol. 2, Issue 3, p. Article 37). Association for Computing Machinery.
52. Cardenas, L., Parajes, K., Zhu, M., & Zhai, S. (2024). AutoHealth: Advanced LLM-Empowered Wearable Personalized Medical Butler for Parkinson’s Disease Management. *2024 IEEE 14th Annual Computing and Communication Workshop and Conference (CCWC)*, 0375–0379. https://doi.org/10.1109/CCWC60891.2024.10427622
53. Casado–Mansilla, D., Gómez–Carmona, O., Fernández–de–Retana, M., Muzzioli, L., Kušar, A., Vandevijvere, S., & López–de–Ipiña, D. (2024). Food Assistant for Consumer Behaviour Change through Citizen Science and AI. *2024 9th International Conference on Smart and Sustainable Technologies (SpliTech)*, 01–06. https://doi.org/10.23919/SpliTech61897.2024.10612651
54. Cascella, M., Semeraro, F., Montomoli, J., Bellini, V., Piazza, O., & Bignami, E. (2024). The Breakthrough of Large Language Models Release for Medical Applications: 1-Year Timeline and Perspectives. *Journal of Medical Systems*, *48*(1), 22. https://doi.org/10.1007/s10916-024-02045-3
55. Casheekar, A., Lahiri, A., Rath, K., Prabhakar, K. S., & Srinivasan, K. (2024). A contemporary review on chatbots, AI-powered virtual conversational agents, ChatGPT: Applications, open challenges and future research directions. *Computer Science Review*, *52*, 100632. https://doi.org/10.1016/j.cosrev.2024.100632
56. Cassar, D. (2023). The Misinformation Threat: A Techno-Governance Approach for Curbing the Fake News of Tomorrow. In *Digit. Gov.: Res. Pract.* (Vol. 4, Issue 4, p. Article 24). Association for Computing Machinery.
57. Castagna, F., Kökciyan, N., Sassoon, I., Parsons, S., & Sklar, E. (2024). Computational Argumentation-based Chatbots: A Survey. In *J. Artif. Int. Res.* (Vol. 80, p. 40). AI Access Foundation.
58. Castro, L. S., Baraças, L., Hashioka, G., Bonadio, C., Hachul, H., Santos-Silva, R., & Poyares, D. (2022). Real world efficacy of a multicomponent CBTi program with chatbot and AI. *Sleep Medicine*, *100*, S135. https://doi.org/10.1016/j.sleep.2022.05.370
59. Castro, O., Mair, J. L., Salamanca-Sanabria, A., Alattas, A., Keller, R., Zheng, S., Jabir, A., Lin, X., Frese, B. F., Lim, C. S., Santhanam, P., Van Dam, R. M., Car, J., Lee, J., Tai, E. S., Fleisch, E., Von Wangenheim, F., Tudor Car, L., Müller-Riemenschneider, F., & Kowatsch, T. (2023). Development of “LvL UP 1.0”: A smartphone-based, conversational agent-delivered holistic lifestyle intervention for the prevention of non-communicable diseases and common mental disorders. *Frontiers in Digital Health*, *5*, 1039171. https://doi.org/10.3389/fdgth.2023.1039171
60. Casu, M., Triscari, S., Battiato, S., Guarnera, L., & Caponnetto, P. (2024). AI Chatbots for Mental Health: A Scoping Review of Effectiveness, Feasibility, and Applications. *Applied Sciences*, *14*(13), 5889. https://doi.org/10.3390/app14135889
61. Cevasco, K. E., Morrison Brown, R. E., Woldeselassie, R., & Kaplan, S. (2024). Patient Engagement with Conversational Agents in Health Applications 2016–2022: A Systematic Review and Meta-Analysis. *Journal of Medical Systems*, *48*(1), 40. https://doi.org/10.1007/s10916-024-02059-x
62. Chan, P. S., Fang, Y., Cheung, D. H., Zhang, Q., Sun, F., Mo, P. K. H., & Wang, Z. (2024). Effectiveness of chatbots in increasing uptake, intention, and attitudes related to any type of vaccination: A systematic review and meta‐analysis. *Applied Psychology: Health and Well-Being*, aphw.12564. https://doi.org/10.1111/aphw.12564
63. Chang, W.-J., Chang, P.-C., & Chang, Y.-H. (2024). The gamification and development of a chatbot to promote oral self-care by adopting behavior change wheel for Taiwanese children. *DIGITAL HEALTH*, *10*, 20552076241256750. https://doi.org/10.1177/20552076241256750
64. Chaudhry, B. M., & Debi, H. R. (2024). User perceptions and experiences of an AI-driven conversational agent for mental health support. *mHealth*, *10*, 22–22. https://doi.org/10.21037/mhealth-23-55
65. Chen, T., Shen, Y., Chen, X., & Zhang, L. (2024). PsyChatbot: A Psychological Counseling Agent Towards Depressed Chinese Population Based on Cognitive Behavioural Therapy. In *ACM Trans. Asian Low-Resour. Lang. Inf. Process.* Association for Computing Machinery. https://doi.org/10.1145/3676962
66. Cheng, Y., Wang, Y., & Lee, J. (2024). Using a Chatbot to Combat Misinformation: Exploring Gratifications, Chatbot Satisfaction and Engagement, and Relationship Quality. *International Journal of Human–Computer Interaction*, 1–13. https://doi.org/10.1080/10447318.2024.2344149
67. Chew, H. S. J. (2022). The Use of Artificial Intelligence–Based Conversational Agents (Chatbots) for Weight Loss: Scoping Review and Practical Recommendations. *JMIR Medical Informatics*, *10*(4), e32578. https://doi.org/10.2196/32578
68. Chin, J., Desai, S., Lin, S. (Cheng-H., & Mejia, S. (2024). Like My Aunt Dorothy: Effects of Conversational Styles on Perceptions, Acceptance and Metaphorical Descriptions of Voice Assistants during Later Adulthood. In *Proc. ACM Hum.-Comput. Interact.* (Vol. 8, Issue CSCW1, p. Article 88). Association for Computing Machinery.
69. Cho, E., Motalebi, N., Sundar, S. S., & Abdullah, S. (2022). Alexa as an Active Listener: How Backchanneling Can Elicit Self-Disclosure and Promote User Experience. In *Proc. ACM Hum.-Comput. Interact.* (Vol. 6, Issue CSCW2, p. Article 273). Association for Computing Machinery.
70. Chow, J. S. F., Blight, V., Brown, M., Glynn, V., Lane, B., Larkin, A., Marshall, S., Matthews, P., Rowles, M., & Warner, B. (2023). Curious thing, an artificial intelligence (AI)-based conversational agent for COVID-19 patient management. *Australian Journal of Primary Health*, *29*(4), 312–318. https://doi.org/10.1071/PY22045
71. Chowdhury, M. N.-U.-R., Haque, A., & Soliman, H. (2023). Chatbots: A Game Changer in mHealth. *2023 Sixth International Symposium on Computer, Consumer and Control (IS3C)*, 362–366. https://doi.org/10.1109/IS3C57901.2023.00103
72. Coleman, T., Till, S., Farao, J., Shandu, L., Khuzwayo, N., Muthelo, L., Mbombi, M., Bopape, M., Heerden, A. van, Mothiba, T., Norris, S., Verdezoto, N., & Densmore, M. (2023). Reconsidering Priorities for Digital Maternal and Child Health: Community-centered Perspectives from South Africa. In *Proc. ACM Hum.-Comput. Interact.* (Vol. 7, Issue CSCW2, p. Article 290). Association for Computing Machinery.
73. Collier, A. F., Hagemann, S., Trinidad, S. B., & Vigil-Hayes, M. (2023). Human-to-Computer Interactivity Features Incorporated Into Behavioral Health mHealth Apps: Systematic Search. *JMIR Formative Research*, *7*, e44926. https://doi.org/10.2196/44926
74. Comulada, W. S., Rezai, R., Sumstine, S., Flores, D. D., Kerin, T., Ocasio, M. A., Swendeman, D., Fernández, M. I., & the Adolescent Trials Network (ATN) CARES Team. (2024). A necessary conversation to develop chatbots for HIV studies: Qualitative findings from research staff, community advisory board members, and study participants. *AIDS Care*, *36*(4), 463–471. https://doi.org/10.1080/09540121.2023.2216926
75. Cook, D., Peters, D., Moradbakhti, L., Su, T., Da Re, M., Schuller, B. W., Quint, J., Wong, E., & Calvo, R. A. (2024). A text-based conversational agent for asthma support: Mixed-methods feasibility study. *DIGITAL HEALTH*, *10*, 20552076241258276. https://doi.org/10.1177/20552076241258276
76. Cooper, H., Nadzri, F. Z. M., Vyas, S., Juhari, R., Ismail, N., Arshat, Z., Rajandiran, D., Markle, L., Calderon, F., Vallance, I., Melendez-Torres, G. J., Facciolà, C., Senesathith, V., Gardner, F., & Lachman, J. M. (2024). A Hybrid Digital Parenting Program Delivered Within the Malaysian Preschool System: Protocol for a Feasibility Study of a Small-Scale Factorial Cluster Randomized Trial. *JMIR Research Protocols*, *13*, e55491. https://doi.org/10.2196/55491
77. Costello, F. J., & Lee, K. C. (2023). Aristotle’s Phronesis as a Philosophical Foundation in Designing the Algorithmic Motivator-Driven Insulating Model (AMOI). In H. Degen, S. Ntoa, & A. Moallem (Eds.), *HCI International 2023 – Late Breaking Papers* (Vol. 14059, pp. 343–355). Springer Nature Switzerland. https://doi.org/10.1007/978-3-031-48057-7_21
78. Cuadra, A., Breuch, J., Estrada, S., Ihim, D., Hung, I., Askaryar, D., Hassanien, M., Fessele, K. L., & Landay, J. A. (2024). Digital Forms for All: A Holistic Multimodal Large Language Model Agent for Health Data Entry. In *Proc. ACM Interact. Mob. Wearable Ubiquitous Technol.* (Vol. 8, Issue 2, p. Article 72). Association for Computing Machinery.
79. Cui, Y., Lee, Y.-J., Jamieson, J., Yamashita, N., & Lee, Y.-C. (2024). Exploring Effects of Chatbot’s Interpretation and Self-disclosure on Mental Illness Stigma. In *Proc. ACM Hum.-Comput. Interact.* (Vol. 8, Issue CSCW1, p. Article 52). Association for Computing Machinery.
80. D, A., M, M., T, P., M, T., Sherin A, P. S., & R, N. E. (2024). Revolutionizing Rural Healthcare in India: AI-Powered Chatbots for Affordable Symptom Analysis and Medical Guidance. *2024 International Conference on Inventive Computation Technologies (ICICT)*, 181–187. https://doi.org/10.1109/ICICT60155.2024.10544758
81. Damij, N., & Bhattacharya, S. (2022). The Role of AI Chatbots in Mental Health Related Public Services in a (Post)Pandemic World: A Review and Future Research Agenda. *2022 IEEE Technology and Engineering Management Conference (TEMSCON EUROPE)*, 152–159. https://doi.org/10.1109/TEMSCONEUROPE54743.2022.9801962
82. De La Rosa-Gómez, A., & Waldherr, K. (2023). Editorial: Highlights in digital mental health 2021/22. *Frontiers in Digital Health*, *4*, 1093375. https://doi.org/10.3389/fdgth.2022.1093375
83. Debbané, A., Lee, K. J., Tse, J., & Law, E. (2023). Learning by Teaching: Key Challenges and Design Implications. In *Proc. ACM Hum.-Comput. Interact.* (Vol. 7, Issue CSCW1, p. Article 68). Association for Computing Machinery.
84. Dehbozorgi, N., & Kunuku, M. T. (2023). Affective Computing: A Topic-Based SER Approach on Collaborative Discussions in Academic Setting. *2023 IEEE Frontiers in Education Conference (FIE)*, 1–7. https://doi.org/10.1109/FIE58773.2023.10342963
85. Denecke, K., Schmid, N., & Nüssli, S. (2022). Implementation of Cognitive Behavioral Therapy in e–Mental Health Apps: Literature Review. *Journal of Medical Internet Research*, *24*(3), e27791. https://doi.org/10.2196/27791
86. Diano, F., Ponticorvo, M., & Sica, L. S. (2022). Mental Health Mobile Apps to Empower Psychotherapy: A Narrative Review. *2022 IEEE International Conference on Metrology for Extended Reality, Artificial Intelligence and Neural Engineering (MetroXRAINE)*, 306–311. https://doi.org/10.1109/MetroXRAINE54828.2022.9967663
87. Dixit, R., Raj, P., Raj, R., & Garg, P. (2024). Your Sanctuary in the Digital Age: A Stress Management Solution Redefining Wellbeing. *2024 International Conference on Intelligent Systems for Cybersecurity (ISCS)*, 1–7. https://doi.org/10.1109/ISCS61804.2024.10581397
88. Dubiel, M., Leiva, L. A., Bongard-Blanchy, K., & Sergeeva, A. (2024). “Hey Genie, You Got Me Thinking About My Menu Choices!”: Impact of Proactive Feedback on User Perception and Reflection in Decision-making Tasks. In *ACM Trans. Comput.-Hum. Interact.* Association for Computing Machinery. https://doi.org/10.1145/3685274
89. Eagle, T., Mehrotra, A., Sharma, A., Zuniga, A., & Whittaker, S. (2022). “Money Doesn’t Buy You Happiness”: Negative Consequences of Using the Freemium Model for Mental Health Apps. In *Proc. ACM Hum.-Comput. Interact.* (Vol. 6, Issue CSCW2, p. Article 265). Association for Computing Machinery.
90. Edhem Yılmaz, İ., Berhuni, M., Özer Özcan, Z., & Doğan, L. (2024). Chatbots talk Strabismus: Can AI become the new patient Educator? *International Journal of Medical Informatics*, *191*, 105592. https://doi.org/10.1016/j.ijmedinf.2024.105592
91. Edison, D., Goswami, S., Janakiraman, V., Almusawi, M., Kuppuraj, T., & Ramesh, A. (2024). An Intensive Role of Chatbot Services in the field of Healthcare. *2024 4th International Conference on Advance Computing and Innovative Technologies in Engineering (ICACITE)*, 21–26. https://doi.org/10.1109/ICACITE60783.2024.10617084
92. Ehrlich, C., Hennelly, S. E., Wilde, N., Lennon, O., Beck, A., Messenger, H., Sergiou, K., & Davies, E. L. (2024). Evaluation of an artificial intelligence enhanced application for student wellbeing: Pilot randomised trial of the Mind Tutor. *International Journal of Applied Positive Psychology*, *9*(1), 435–454. APA PsycInfo®. https://doi.org/10.1007/s41042-023-00133-2
93. Eighth International Symposium on hereditary breast and ovarian cancer: May 4–7, 2021 – Virtual Edition. (2022). *Familial Cancer*, *21*(3), 255–288. https://doi.org/10.1007/s10689-021-00273-x
94. El Ayadi, A. M., Singh, P., Duggal, M., Kumar, V., Kaur, J., Sharma, P., Vosburg, K. B., & Diamond-Smith, N. G. (2023). Feasibility and acceptability of Saheli, a WhatsApp Chatbot, on COVID-19 vaccination among pregnant and breastfeeding women in rural North India. *BMJ Innovations*, *9*(4), 195–206. https://doi.org/10.1136/bmjinnov-2022-001012
95. Emezue, C., Karnik, N. S., Reeder, B., Schoeny, M., Layfield, R., Zarling, A., & Julion, W. (2023). A Technology-Enhanced Intervention for Violence and Substance Use Prevention Among Young Black Men: Protocol for Adaptation and Pilot Testing. *JMIR Research Protocols*, *12*, e43842. https://doi.org/10.2196/43842
96. Emmer De Albuquerque Green, C. (2024). Defining responsible use of AI chatbots in social care for older adults. *Nature Aging*, *4*(6), 745–747. https://doi.org/10.1038/s43587-024-00636-w
97. Entenberg, G. A., Dosovitsky, G., Aghakhani, S., Mostovoy, K., Carre, N., Marshall, Z., Benfica, D., Mizrahi, S., Testerman, A., Rousseau, A., Lin, G., & Bunge, E. L. (2023). User experience with a parenting chatbot micro intervention. *Frontiers in Digital Health*, *4*, 989022. https://doi.org/10.3389/fdgth.2022.989022
98. Entenberg, G. A., Mizrahi, S., Walker, H., Aghakhani, S., Mostovoy, K., Carre, N., Marshall, Z., Dosovitsky, G., Benfica, D., Rousseau, A., Lin, G., & Bunge, E. L. (2023). AI-based chatbot micro-intervention for parents: Meaningful engagement, learning, and efficacy. *Frontiers in Psychiatry*, *14*, 1080770. https://doi.org/10.3389/fpsyt.2023.1080770
99. Epstein, D. A., Liu, F., Monroy-Hernández, A., & Wang, D. (2022). Revisiting Piggyback Prototyping: Examining Benefits and Tradeoffs in Extending Existing Social Computing Systems. In *Proc. ACM Hum.-Comput. Interact.* (Vol. 6, Issue CSCW2, p. Article 456). Association for Computing Machinery.
100. Escobar-Viera, C. G., Porta, G., Coulter, R. W. S., Martina, J., Goldbach, J., & Rollman, B. L. (2023). A chatbot-delivered intervention for optimizing social media use and reducing perceived isolation among rural-living LGBTQ+ youth: Development, acceptability, usability, satisfaction, and utility. *Internet Interventions*, *34*, 100668. https://doi.org/10.1016/j.invent.2023.100668
101. Fabian, K. E., Foster, K. T., Chwastiak, L., Turner, M., & Wagenaar, B. H. (2023). Adapting a transdiagnostic digital mental health intervention for use among immigrant and refugee youth in Seattle: A human-centered design approach. *Translational Behavioral Medicine*, *13*(11), 867–875. https://doi.org/10.1093/tbm/ibad041
102. Fang, A., & Zhu, H. (2022). Matching for Peer Support: Exploring Algorithmic Matching for Online Mental Health Communities. In *Proc. ACM Hum.-Comput. Interact.* (Vol. 6, Issue CSCW2, p. Article 311). Association for Computing Machinery.
103. Farrand, P., Raue, P. J., Ward, E., Repper, D., & Areán, P. (2024). Use and Engagement With Low-Intensity Cognitive Behavioral Therapy Techniques Used Within an App to Support Worry Management: Content Analysis of Log Data. *JMIR mHealth and uHealth*, *12*, e47321. https://doi.org/10.2196/47321
104. Forman-Hoffman, V. L., Pirner, M. C., Flom, M., Kirvin-Quamme, A., Durden, E., Kissinger, J. A., & Robinson, A. (2023). Engagement, Satisfaction, and Mental Health Outcomes Across Different Residential Subgroup Users of a Digital Mental Health Relational Agent: Exploratory Single-Arm Study. *JMIR Formative Research*, *7*, e46473. https://doi.org/10.2196/46473
105. Formosa, P., Kashyap, B., & Sahebi, S. (2024). Generative AI and the Future of Democratic Citizenship. In *Digit. Gov.: Res. Pract.* Association for Computing Machinery. https://doi.org/10.1145/3674844
106. Garimella, B. S., Garlapati, H. S., Choul, S., Cherukuri, R., & Lanke, P. (2024). Advancing Healthcare Accessibility: Development of an AI-Driven Multimodal Chatbot. *2023 4th International Conference on Intelligent Technologies (CONIT)*, 1–10. https://doi.org/10.1109/CONIT61985.2024.10626795
107. Ghanem, S. I., Hamshary, E., Matar, L., & Morsy, A. (2024). Enhancing Healthcare Service with Firebase Integration and Intelligent Chatbot Deployment. *2024 Intelligent Methods, Systems, and Applications (IMSA)*, 550–555. https://doi.org/10.1109/IMSA61967.2024.10652750
108. Ghanem, Y. K., Rouhi, A. D., Al-Houssan, A., Saleh, Z., Moccia, M. C., Joshi, H., Dumon, K. R., Hong, Y., Spitz, F., Joshi, A. R., & Kwiatt, M. (2024). Dr. Google to Dr. ChatGPT: Assessing the content and quality of artificial intelligence-generated medical information on appendicitis. *Surgical Endoscopy*, *38*(5), 2887–2893. https://doi.org/10.1007/s00464-024-10739-5
109. Ghim, J.-L., & Ahn, S. (2023). Transforming clinical trials: The emerging roles of large language models. *Translational and Clinical Pharmacology*, *31*(3), 131. https://doi.org/10.12793/tcp.2023.31.e16
110. Gilson, A., Chen, Q., Singer, M., Xu, H., & Adelman, R. A. (2023). Large language models and the retina: A review of current applications and future directions. *Journal of Retina-Vitreous*, *32*(4), 225. https://doi.org/10.37845/ret.vit.2023.32.38
111. Gkountara, D. N., & Prasad, R. (2022). A review of Artificial Intelligence in Foreign Language Learning. *2022 25th International Symposium on Wireless Personal Multimedia Communications (WPMC)*, 134–139. https://doi.org/10.1109/WPMC55625.2022.10014767
112. Goh, M., Jeong, H., Yoo, J. H., & Han, O. (2023). Self-Disclosure in Digital Healthcare: Enhancing User Engagement. *2023 IEEE International Conference on Agents (ICA)*, 63–68. https://doi.org/10.1109/ICA58824.2023.00020
113. Golden, A. H., Gabriel, M. H., Russo, J., Price, M., Ruhmel, S., Nilsson, A., Delong, P. S., Jelsma, J., & Carty, M. (2023). Let’s talk about it: An exploration of the comparative use of three different digital platforms to gather patient-reported outcome measures. *Journal of Patient-Reported Outcomes*, *7*(1), 130. https://doi.org/10.1186/s41687-023-00666-9
114. Gomaa, S., Posey, J., Bashir, B., Basu Mallick, A., Vanderklok, E., Schnoll, M., Zhan, T., & Wen, K.-Y. (2023). Feasibility of a Text Messaging–Integrated and Chatbot-Interfaced Self-Management Program for Symptom Control in Patients With Gastrointestinal Cancer Undergoing Chemotherapy: Pilot Mixed Methods Study. *JMIR Formative Research*, *7*, e46128. https://doi.org/10.2196/46128
115. Goonesekera, Y., & Donkin, L. (2022). A Cognitive Behavioral Therapy Chatbot (Otis) for Health Anxiety Management: Mixed Methods Pilot Study. *JMIR Formative Research*, *6*(10), e37877. https://doi.org/10.2196/37877
116. Gordon, E. B., Maxfield, C., French, R., Fish, L. J., Romm, J., Barre, E., Kinne, E., Peterson, R., & Grimm, L. J. (2024). Large Language Model Use in Radiology Residency Applications: Unwelcomed but Inevitable. *Journal of the American College of Radiology*, S1546144024007683. https://doi.org/10.1016/j.jacr.2024.08.027
117. Gordon, E. J., Gacki-Smith, J., Gooden, M. J., Waite, P., Yacat, R., Abubakari, Z. R., Duquette, D., Agrawal, A., Friedewald, J., Savage, S. K., Cooper, M., Gilbert, A., Muhammad, L. N., & Wicklund, C. (2024). Development of a culturally targeted chatbot to inform living kidney donor candidates of African ancestry about APOL1 genetic testing: A mixed methods study. *Journal of Community Genetics*, *15*(2), 205–216. https://doi.org/10.1007/s12687-024-00698-8
118. Green, R. W., & Castro, H. (2024). Transforming Otolaryngology—Head and Neck Surgery. *Otolaryngologic Clinics of North America*, *57*(5), 909–918. https://doi.org/10.1016/j.otc.2024.04.003
119. Guo, N., Luk, T. T., Wu, Y., Guo, Z., Chu, J. C. L., Cheung, Y. T. D., Chan, C. H. H., Kwok, T. T. O., Wong, V. Y. L., Wong, C. K. H., Lee, J. J., Kwok, Y. K., Viswanath, K., Lam, T. H., & Wang, M. (2023). Effect of mobile interventions with nicotine replacementtherapy sampling on long-term smoking cessation incommunity smokers: A pragmatic randomized clinical trial. *Tobacco Induced Diseases*, *21*(March), 1–13. https://doi.org/10.18332/tid/160168
120. Gurnani, B., & Kaur, K. (2024). Leveraging ChatGPT for ophthalmic education: A critical appraisal. *European Journal of Ophthalmology*, *34*(2), 323–327. https://doi.org/10.1177/11206721231215862
121. Hallsworth, J. E., Udaondo, Z., Pedrós‐Alió, C., Höfer, J., Benison, K. C., Lloyd, K. G., Cordero, R. J. B., De Campos, C. B. L., Yakimov, M. M., & Amils, R. (2023). Scientific novelty beyond the experiment. *Microbial Biotechnology*, *16*(6), 1131–1173. https://doi.org/10.1111/1751-7915.14222
122. Han, G., Liu, W., Huang, X., & Borsari, B. (2024). Chain-of-Interaction: Enhancing Large Language Models for Psychiatric Behavior Understanding by Dyadic Contexts. *2024 IEEE 12th International Conference on Healthcare Informatics (ICHI)*, 392–401. https://doi.org/10.1109/ICHI61247.2024.00057
123. Han, R., Todd, A., Wardak, S., Partridge, S. R., & Raeside, R. (2023). Feasibility and acceptability of chatbots for nutrition and physical activity health promotion among adolescents: Systematic scoping review with adolescent consultation. *JMIR Human Factors*, *10*, 12. APA PsycInfo®. https://doi.org/10.2196/43227
124. Haque, A., Chowdhury, M. N.-U.-R., & Soliman, H. (2023). Transforming Chronic Disease Management with Chatbots: Key Use Cases for Personalized and Cost-effective Care. *2023 Sixth International Symposium on Computer, Consumer and Control (IS3C)*, 367–370. https://doi.org/10.1109/IS3C57901.2023.00104
125. Haque, M. R., & Rubya, S. (2022). “For an App Supposed to Make Its Users Feel Better, It Sure is a Joke”—An Analysis of User Reviews of Mobile Mental Health Applications. In *Proc. ACM Hum.-Comput. Interact.* (Vol. 6, Issue CSCW2, p. Article 421). Association for Computing Machinery.
126. Hardy, J., Geier, C., Vargas, S., Doll, R., & Howard, A. L. (2022). LGBTQ Futures and Participatory Design: Investigating Visibility, Community, and the Future of Future Workshops. In *Proc. ACM Hum.-Comput. Interact.* (Vol. 6, Issue CSCW2, p. Article 525). Association for Computing Machinery.
127. Hartinger Pena, S. M., Mäusezahl, D., Jäggi, L., Aguilar, L., Alvarado Llatance, M., Castellanos, A., Huaylinos Bustamante, M.-L., Hinckley, K., Charles McCoy, D., Zhang, C., & Fink, G. (2023). Digital Support Systems to Improve Child Health and Development in Peru: Protocol for a Randomized Controlled Trial. *JMIR Research Protocols*, *12*, e50371. https://doi.org/10.2196/50371
128. He, L., Basar, E., Krahmer, E., Wiers, R., & Antheunis, M. (2024). Effectiveness and User Experience of a Smoking Cessation Chatbot: Mixed Methods Study Comparing Motivational Interviewing and Confrontational Counseling. *Journal of Medical Internet Research*, *26*, e53134. https://doi.org/10.2196/53134
129. He, L., Basar, E., Wiers, R. W., Antheunis, M. L., & Krahmer, E. (2022). Can chatbots help to motivate smoking cessation? A study on the effectiveness of motivational interviewing on engagement and therapeutic alliance. *BMC Public Health*, *22*(1), 726. https://doi.org/10.1186/s12889-022-13115-x
130. Hellstrom, W., Singh, D., Brinkley, G., Trost, L., Ziegelmann, M., Brock, G., Khera, M., & Mulhall, J. (2024). Can Artificial Intelligence Generate a Superior Introduction to a Systematic Review? *The Journal of Sexual Medicine*, *21*(Supplement_1), qdae001.021. https://doi.org/10.1093/jsxmed/qdae001.021
131. Hemkiran, S., War, M. M., Aadhithiyan, K. S., & Kabilan, K. (2024). Web-Based Patient Health Management System with Doctor Recommendations and Medicine Alternatives using Machine Learning. *2024 International Conference on Smart Systems for Electrical, Electronics, Communication and Computer Engineering (ICSSEECC)*, 68–73. https://doi.org/10.1109/ICSSEECC61126.2024.10649525
132. Herrera, L., Gjøsæter, T., Majchrzak, T. A., & Thapa, D. (2024). Signals of Transition in Support Systems: A Study of the use of Social Media Analytics in Crisis Management: A Study of the use of Social Media Analytics in Crisis Management. In *Trans. Soc. Comput.* Association for Computing Machinery. https://doi.org/10.1145/3649474
133. Heston, T. F. (2023). Safety of Large Language Models in Addressing Depression. *Cureus*. https://doi.org/10.7759/cureus.50729
134. Huang, J., Ma, N. F., Rivera, V. A., Somani, T., Lee, P. Y. K., Mcgrenere, J., & Yoon, D. (2024). Design Tensions in Online Freelancing Platforms: Using Speculative Participatory Design to Support Freelancers’ Relationships with Clients. In *Proc. ACM Hum.-Comput. Interact.* (Vol. 8, Issue CSCW1, p. Article 209). Association for Computing Machinery.
135. Huang, S., Fu, F., Yang, K., Zhang, K., & Yang, F. (2024). Empowerment of Large Language Models in Psychological Counseling through Prompt Engineering. *2024 IEEE 4th International Conference on Software Engineering and Artificial Intelligence (SEAI)*, 220–225. https://doi.org/10.1109/SEAI62072.2024.10674052
136. Hueso, M., Álvarez, R., Marí, D., Ribas-Ripoll, V., Lekadir, K., & Vellido, A. (2023). Is generative artificial intelligence the next step toward a personalized hemodialysis? *Revista de Investigacin Clnica*, *75*(6), 11703. https://doi.org/10.24875/RIC.23000162
137. ICIMH 2022 Abstracts. (2022). *Global Advances in Health and Medicine*, *11*, 2164957X2210965. https://doi.org/10.1177/2164957X221096590
138. Iniesto, F., Coughlan, T., Lister, K., Devine, P., Freear, N., Greenwood, R., Holmes, W., Kenny, I., McLeod, K., & Tudor, R. (2023). Creating ‘a Simple Conversation’: Designing a Conversational User Interface to Improve the Experience of Accessing Support for Study. In *ACM Trans. Access. Comput.* (Vol. 16, Issue 1, p. Article 6). Association for Computing Machinery.
139. Inkster, B., Kadaba, M., & Subramanian, V. (2023). Understanding the impact of an AI-enabled conversational agent mobile app on users’ mental health and wellbeing with a self-reported maternal event: A mixed method real-world data mHealth study. *Frontiers in Global Women’s Health*, *4*, 1084302. https://doi.org/10.3389/fgwh.2023.1084302
140. Invited Speaker Presentation Abstracts. (2023). *International Journal of Rheumatic Diseases*, *26*(S1), 4–25. https://doi.org/10.1111/1756-185X.14502
141. Islam, R., Sarkar, R., & Sarkar, R. R. (2024). Integrated Blood Donation System with Smart Vending Machines. *2024 3rd International Conference on Advancement in Electrical and Electronic Engineering (ICAEEE)*, 1–5. https://doi.org/10.1109/ICAEEE62219.2024.10561675
142. Jia, C., Lam, M. S., Mai, M. C., Hancock, J. T., & Bernstein, M. S. (2024). Embedding Democratic Values into Social Media AIs via Societal Objective Functions. In *Proc. ACM Hum.-Comput. Interact.* (Vol. 8, Issue CSCW1, p. Article 163). Association for Computing Machinery.
143. Jiang, Y. (2024). The Applications of Large Language Models in Emergency Management. *2024 IEEE 6th Advanced Information Management, Communicates, Electronic and Automation Control Conference (IMCEC)*, 199–202. https://doi.org/10.1109/IMCEC59810.2024.10575031
144. Jiang, Z., Rashik, M., Panchal, K., Jasim, M., Sarvghad, A., Riahi, P., DeWitt, E., Thurber, F., & Mahyar, N. (2023). CommunityBots: Creating and Evaluating A Multi-Agent Chatbot Platform for Public Input Elicitation. In *Proc. ACM Hum.-Comput. Interact.* (Vol. 7, Issue CSCW1, p. Article 36). Association for Computing Machinery.
145. Jin, Y., Cai, W., Chen, L., Dai, Y., & Jiang, T. (2023). Understanding Disclosure and Support for Youth Mental Health in Social Music Communities. In *Proc. ACM Hum.-Comput. Interact.* (Vol. 7, Issue CSCW1, p. Article 153). Association for Computing Machinery.
146. Jonassaint, C. R., Lalama, C. M., O’Brien, J. A., Badawy, S. M., Hamm, M. E., Stinson, J., Lalloo, C., Carroll, C. P., Saraf, S. L., Gordeuk, V. R., Cronin, R., Shah, N., Lanzkron, S. M., Liles, D. K., Saint-Jean, L., DeBaun, M. R., Trimnell, C., Bailey, L., Lawrence, R. H., … Abebe, K. (2023). A Randomized Clinical Trial Testing the Effect of Digital Behavioral Interventions on Pain and Mental Health Among Adults with Sickle Cell Disease. *Blood*, *142*(Supplement 1), 789–789. https://doi.org/10.1182/blood-2023-186763
147. K, R., Gupta, M., Abdul-Zahra, D. S., Subhashini, K., Maiti, N., & Chawla, R. (2023). Healthcare Chatbots With Nlp and Cybersecurity: Safeguarding Patient Data in the Cloud. *2023 International Conference on Artificial Intelligence for Innovations in Healthcare Industries (ICAIIHI)*, 1–7. https://doi.org/10.1109/ICAIIHI57871.2023.10489713
148. Kallivalappil, N., D’souza, K., Deshmukh, A., Kadam, C., & Sharma, N. (2023). Empath.ai: A Context-Aware Chatbot for Emotional Detection and Support. *2023 14th International Conference on Computing Communication and Networking Technologies (ICCCNT)*, 1–7. https://doi.org/10.1109/ICCCNT56998.2023.10306584
149. Karhiy, M., Sagar, M., Antoni, M., Loveys, K., & Broadbent, E. (2023). Mindfulness based stress reduction: A randomised trial of a virtual human, teletherapy, and a chatbot. *2023 11th International Conference on Affective Computing and Intelligent Interaction Workshops and Demos (ACIIW)*, 1–7. https://doi.org/10.1109/ACIIW59127.2023.10388195
150. Karkosz, S., Szymański, R., Sanna, K., & Michałowski, J. (2024). Effectiveness of a Web-based and Mobile Therapy Chatbot on Anxiety and Depressive Symptoms in Subclinical Young Adults: Randomized Controlled Trial. *JMIR Formative Research*, *8*, e47960. https://doi.org/10.2196/47960
151. Karusala, N., Yan, S., Rajkumar, N., G, V., & Anderson, R. (2023). Speculating with Care: Worker-centered Perspectives on Scale in a Chat-based Health Information Service. In *Proc. ACM Hum.-Comput. Interact.* (Vol. 7, Issue CSCW2, p. Article 361). Association for Computing Machinery.
152. Kashyap, N., Sebastian, A. T., Lynch, C., Jansons, P., Maddison, R., Dingler, T., & Oldenburg, B. (2024). Engagement With Conversational Agent–Enabled Interventions in Cardiometabolic Disease Management: Protocol for a Systematic Review. *JMIR Research Protocols*, *13*, e52973. https://doi.org/10.2196/52973
153. Kaur, J., Sharma, P., Kumar, V., Duggal, M., Diamond-Smith, N. G., Ayadi, A. E., Vosburg, K., & Singh, P. (2024). Exploring the Role of Chatbots in Tackling COVID-19 Vaccine Hesitancy among Pregnant and Breastfeeding Women in Rural Northern India. In *Proc. ACM Hum.-Comput. Interact.* (Vol. 8, Issue CSCW1, p. Article 55). Association for Computing Machinery.
154. Kaywan, P., Ahmed, K., Ibaida, A., Miao, Y., & Gu, B. (2023). Early detection of depression using a conversational AI bot: A non-clinical trial. *PLOS ONE*, *18*(2), e0279743. https://doi.org/10.1371/journal.pone.0279743
155. Kettle, L., & Lee, Y.-C. (2024). User Experiences of Well-Being Chatbots. *Human Factors: The Journal of the Human Factors and Ergonomics Society*, *66*(6), 1703–1723. https://doi.org/10.1177/00187208231162453
156. Keyvan, K., & Huang, J. X. (2022). How to Approach Ambiguous Queries in Conversational Search: A Survey of Techniques, Approaches, Tools, and Challenges. In *ACM Comput. Surv.* (Vol. 55, Issue 6, p. Article 129). Association for Computing Machinery.
157. Khosravi, M., & Azar, G. (2024). Factors influencing patient engagement in mental health chatbots: A thematic analysis of findings from a systematic review of reviews. *DIGITAL HEALTH*, *10*, 20552076241247983. https://doi.org/10.1177/20552076241247983
158. Kim, I., Ko, M., Park, J., Moon, S. W., Jung, G., Lim, Y., & Lee, U. (2022). Social-Spiritual Face: Designing Social Reading Support for Spiritual Well-being. In *Proc. ACM Hum.-Comput. Interact.* (Vol. 6, Issue CSCW2, p. Article 262). Association for Computing Machinery.
159. Kim, Y., Kang, Y., Kim, B., Kim, J., & Kim, G. H. (2024). Exploring the role of engagement and adherence in chatbot-based cognitive training for older adults: Memory function and mental health outcomes. *Behaviour & Information Technology*, 1–13. https://doi.org/10.1080/0144929X.2024.2362406
160. Kovačević, N., Holz, C., Gross, M., & Wampfler, R. (2024). The Personality Dimensions GPT-3 Expresses During Human-Chatbot Interactions. In *Proc. ACM Interact. Mob. Wearable Ubiquitous Technol.* (Vol. 8, Issue 2, p. Article 61). Association for Computing Machinery.
161. Kumar, T., Kait, R., Ankita, & Rani, S. (2023). Possibilities and Pitfalls of Generative Pre-Trained Transformers in Healthcare. *2023 International Conference on Advanced Computing & Communication Technologies (ICACCTech)*, 37–44. https://doi.org/10.1109/ICACCTech61146.2023.00016
162. Kwok, I., Freedman, M., Kamsickas, L., Lattie, E. G., Yang, D., & Moskowitz, J. T. (2024). The SoCAP (Social Communication, Affiliation, and Presence) Taxonomy of Social Features: Scoping Review of Commercially Available eHealth Apps. *Journal of Medical Internet Research*, *26*, e49714. https://doi.org/10.2196/49714
163. Kwon, M., Lee, E., Hong, S., & Chun, S. A. (2024). Understanding the Determinants of Digital Innovation in California Local Governments: Digital Inequality and Agency Assignment Perspectives. In *Digit. Gov.: Res. Pract.* (Vol. 5, Issue 3, p. Article 25). Association for Computing Machinery.
164. Lahat, A., Sharif, K., Zoabi, N., Shneor Patt, Y., Sharif, Y., Fisher, L., Shani, U., Arow, M., Levin, R., & Klang, E. (2024). Assessing Generative Pretrained Transformers (GPT) in Clinical Decision-Making: Comparative Analysis of GPT-3.5 and GPT-4. *Journal of Medical Internet Research*, *26*, e54571. https://doi.org/10.2196/54571
165. Lai, Y.-J., Lee, Y.-C., Chang, C.-C., Dai, W.-T., & Chen, Y.-Y. (2024). Exploring the Role of Mom’s Chat Groups in the Messaging App: Enhancing Support and Empowerment for Stay-At-Home Mothers. In *Proc. ACM Hum.-Comput. Interact.* (Vol. 8, Issue GROUP, p. Article 3). Association for Computing Machinery.
166. Laker, B., & Currell, E. (2024). ChatGPT: A novel AI assistant for healthcare messaging—a commentary on its potential in addressing patient queries and reducing clinician burnout. *BMJ Leader*, *8*(2), 147–148. https://doi.org/10.1136/leader-2023-000844
167. Larkin, C., Djamasbi, S., Boudreaux, E. D., Varzgani, F., Garner, R., Siddique, M., Pietro, J., & Tulu, B. (2023). ReachCare Mobile Apps for Patients Experiencing Suicidality in the Emergency Department: Development and Usability Testing Using Mixed Methods. *JMIR Formative Research*, *7*, e41422. https://doi.org/10.2196/41422
168. Lau-Min, K. S., Marini, J., Shah, N., Pucci, D., Blauch, A., Cambareri, C., Mooney, B., Johnston, C., Schumacher, R. P., White, K., Gabriel, P. E., Rosin, R., Jacobs, L. A., & Shulman, L. N. (2022). An augmented intelligence mobile phone chatbot for medication adherence and toxicity management among patients with gastrointestinal cancers on capecitabine. *Journal of Clinical Oncology*, *40*(28_suppl), 424–424. https://doi.org/10.1200/JCO.2022.40.28_suppl.424
169. Law, A. J., Hu, R., Alazraki, L., Gopalan, A., Polydorou, N., & Edalat, A. (2022). A Multilingual Virtual Guide for Self-Attachment Technique. *2022 IEEE 4th International Conference on Cognitive Machine Intelligence (CogMI)*, 107–116. https://doi.org/10.1109/CogMI56440.2022.00025
170. Lawson McLean, A., Wu, Y., Lawson McLean, A. C., & Hristidis, V. (2024). Large language models as decision aids in neuro-oncology: A review of shared decision-making applications. *Journal of Cancer Research and Clinical Oncology*, *150*(3), 139. https://doi.org/10.1007/s00432-024-05673-x
171. Laymouna, M., Ma, Y., Lessard, D., Engler, K., Therrien, R., Schuster, T., Vicente, S., Achiche, S., El Haj, M. N., Lemire, B., Kawaiah, A., & Lebouché, B. (2024). Needs-Assessment for an Artificial Intelligence-Based Chatbot for Pharmacists in HIV Care: Results from a Knowledge–Attitudes–Practices Survey. *Healthcare*, *12*(16), 1661. https://doi.org/10.3390/healthcare12161661
172. Lee, P. Y. K., Ma, N. F., Kim, I.-J., & Yoon, D. (2023). Speculating on Risks of AI Clones to Selfhood and Relationships: Doppelganger-phobia, Identity Fragmentation, and Living Memories. In *Proc. ACM Hum.-Comput. Interact.* (Vol. 7, Issue CSCW1, p. Article 91). Association for Computing Machinery.
173. Lee, T.-C., Staller, K., & Kuo, B. (2024). Reply. *Gastroenterology*, *166*(1), 221–222. https://doi.org/10.1053/j.gastro.2023.10.009
174. Lee, U., Jung, G., Park, S., Ma, E.-Y., Kim, H., Lee, Y., & Noh, Y. (2023). Data-driven Digital Therapeutics Analytics. *2023 IEEE International Conference on Big Data and Smart Computing (BigComp)*, 386–388. https://doi.org/10.1109/BigComp57234.2023.00093
175. Leo, A. J., Schuelke, M. J., Hunt, D. M., Metzler, J. P., Miller, J. P., Areán, P. A., Armbrecht, M. A., & Cheng, A. L. (2022). A Digital Mental Health Intervention in an Orthopedic Setting for Patients With Symptoms of Depression and/or Anxiety: Feasibility Prospective Cohort Study. *JMIR Formative Research*, *6*(2), e34889. https://doi.org/10.2196/34889
176. Li, H., Zhang, R., Lee, Y.-C., Kraut, R. E., & Mohr, D. C. (2023). Systematic review and meta-analysis of AI-based conversational agents for promoting mental health and well-being. *Npj Digital Medicine*, *6*(1), 236. https://doi.org/10.1038/s41746-023-00979-5
177. Liang, K.-H., Shi, W., Oh, Y. J., Wang, H.-C., Zhang, J., & Yu, Z. (2024). Dialoging Resonance in Human-Chatbot Conversation: How Users Perceive and Reciprocate Recommendation Chatbot’s Self-Disclosure Strategy. In *Proc. ACM Hum.-Comput. Interact.* (Vol. 8, Issue CSCW1, p. Article 200). Association for Computing Machinery.
178. Liao, X. Y., Wang, P. H., & Chang, I.-C. (2024). Exploring the Impact of a ChatGPT-Based Interactive System on Enhancing the Knowledge and Attitudes Towards Advance Care Planning in Older Adults. *2024 IEEE 48th Annual Computers, Software, and Applications Conference (COMPSAC)*, 1488–1489. https://doi.org/10.1109/COMPSAC61105.2024.00202
179. Lim, B., Seth, I., Cuomo, R., Kenney, P. S., Ross, R. J., Sofiadellis, F., Pentangelo, P., Ceccaroni, A., Alfano, C., & Rozen, W. M. (2024). Can AI Answer My Questions? Utilizing Artificial Intelligence in the Perioperative Assessment for Abdominoplasty Patients. *Aesthetic Plastic Surgery*. https://doi.org/10.1007/s00266-024-04157-0
180. Lin, X., Martinengo, L., Jabir, A. I., Ho, A. H. Y., Car, J., Atun, R., & Tudor Car, L. (2023). Scope, Characteristics, Behavior Change Techniques, and Quality of Conversational Agents for Mental Health and Well-Being: Systematic Assessment of Apps. *Journal of Medical Internet Research*, *25*, e45984. https://doi.org/10.2196/45984
181. Lin, Y., & Gatica-Perez, D. (2023). Characterizing Swiss Alpine Lakes: From Wikipedia to Citizen Science. In *ACM J. Comput. Sustain. Soc.* (Vol. 1, Issue 2, p. Article 13). Association for Computing Machinery.
182. Liu, I., Xiao, Y., Liu, F., Wu, S., & Ni, S. (2022). Assessing the Effectiveness of Using Chatbots for Positive Psychological Intervention: A Randomized Control Study. *Proceedings of the Tenth International Symposium of Chinese CHI*, 227–234. https://doi.org/10.1145/3565698.3565789
183. Liu, J., & Wang, H. (2024). Exploration on Artificial Intelligence Based on ChatGPT. *2024 5th International Seminar on Artificial Intelligence, Networking and Information Technology (AINIT)*, 6–9. https://doi.org/10.1109/AINIT61980.2024.10581750
184. Liu, Y., Mayfield, R., & Huang, Y. (2023). Discovering the Hidden Facts of User-Dispatcher Interactions via Text-based Reporting Systems for Community Safety. In *Proc. ACM Hum.-Comput. Interact.* (Vol. 7, Issue CSCW1, p. Article 126). Association for Computing Machinery.
185. Luetke Lanfer, H., Reifegerste, D., Berg, A., Memenga, P., Baumann, E., Weber, W., Geulen, J., Müller, A., Hahne, A., & Weg-Remers, S. (2023). Understanding Trust Determinants in a Live Chat Service on Familial Cancer: Qualitative Triangulation Study With Focus Groups and Interviews in Germany. *Journal of Medical Internet Research*, *25*, e44707. https://doi.org/10.2196/44707
186. Lyzwinski, L. N., Elgendi, M., & Menon, C. (2023). Conversational Agents and Avatars for Cardiometabolic Risk Factors and Lifestyle-Related Behaviors: Scoping Review. *JMIR mHealth and uHealth*, *11*, e39649. https://doi.org/10.2196/39649
187. M, N., A, S., R, A., Kumar M, T., S, H., & Prakash M, V. (2024). HealthBot Analytics: Optimizing Healthcare Efficiency Through Intelligent Integration. *2024 International Conference on Advances in Data Engineering and Intelligent Computing Systems (ADICS)*, 1–7. https://doi.org/10.1109/ADICS58448.2024.10533512
188. Ma, Y., Achiche, S., Pomey, M.-P., Paquette, J., Adjtoutah, N., Vicente, S., Engler, K., MARVIN chatbots Patient Expert Committee, Laymouna, M., Lessard, D., Lemire, B., Asselah, J., Therrien, R., Osmanlliu, E., Zawati, M. H., Joly, Y., & Lebouché, B. (2024). Adapting and Evaluating an AI-Based Chatbot Through Patient and Stakeholder Engagement to Provide Information for Different Health Conditions: Master Protocol for an Adaptive Platform Trial (the MARVIN Chatbots Study). *JMIR Research Protocols*, *13*, e54668. https://doi.org/10.2196/54668
189. Magee, M. F., Nassar, C. M., Dunlea, R., James, B., Montero, A. R., Sack, P., Tweedt, A., Vetal, D., & Cobb, N. K. (2022). Engagement, Satisfaction, and Preliminary Impact of a Personalized Diabetes Chatbot in a Diverse Cohort of Adults with Type 2 Diabetes. *Diabetes*, *71*(Supplement_1), 330-OR. https://doi.org/10.2337/db22-330-OR
190. Mahbub, S. B., Islam, N., Surem, M. H., Arefin, A., Shabab, M. R., & Islam, A. (2024). GorbhoKotha: A mHealth App for Maternal Support in Bangladesh Utilizing User-Centered Design Principles. *2024 International Congress on Human-Computer Interaction, Optimization and Robotic Applications (HORA)*, 1–6. https://doi.org/10.1109/HORA61326.2024.10550569
191. Maher, C., Singh, B., Wylde, A., & Chastin, S. (2024). Virtual health assistants: A grand challenge in health communications and behavior change. *Frontiers in Digital Health*, *6*, 1418695. https://doi.org/10.3389/fdgth.2024.1418695
192. Mahmood, A., & Huang, C.-M. (2024). Gender Biases in Error Mitigation by Voice Assistants. In *Proc. ACM Hum.-Comput. Interact.* (Vol. 8, Issue CSCW1, p. Article 60). Association for Computing Machinery.
193. Mak, W. W. S., Ng, S. M., & Leung, F. H. T. (2023). A Web-Based Stratified Stepped Care Platform for Mental Well-being (TourHeart+): User-Centered Research and Design. *JMIR Formative Research*, *7*, e38504. https://doi.org/10.2196/38504
194. Malkin, N., Wagner, D., & Egelman, S. (2022). Can Humans Detect Malicious Always-Listening Assistants? A Framework for Crowdsourcing Test Drives. In *Proc. ACM Hum.-Comput. Interact.* (Vol. 6, Issue CSCW2, p. Article 500). Association for Computing Machinery.
195. Manzi, S. F. (2023). A Life Less Ordinary. *The Journal of Pediatric Pharmacology and Therapeutics*, *28*(6), 480–489. https://doi.org/10.5863/1551-6776-28.6.480
196. Mariani, M. M., Hashemi, N., & Wirtz, J. (2023). Artificial intelligence empowered conversational agents: A systematic literature review and research agenda. *Journal of Business Research*, *161*, 113838. https://doi.org/10.1016/j.jbusres.2023.113838
197. Mariappan, R., Manjunath, L., Ramachandran, G., Porkodi, M., & Sheela, T. (2022). Super Artificial Intelligence Medical Care Systems with IoT Wireless Sensor. *2022 IEEE International Conference on Distributed Computing and Electrical Circuits and Electronics (ICDCECE)*, 1–4. https://doi.org/10.1109/ICDCECE53908.2022.9792895
198. Marrs, J. C., Orlando, S. T., Saseen, J. J., Novins-Montague, S., Sandy, L. C., Waughtal, J., Glorioso, T. J., & Ho, P. M. (2023). Description of patient questions received by clinical pharmacists in the Nudge Study. *American Journal of Health-System Pharmacy*, *80*(18), 1247–1254. https://doi.org/10.1093/ajhp/zxad139
199. Martínez-Ezquerro, J. D. (2023). Response to: Impact of ChatGPT and Artificial Intelligence in the Contemporary Medical Landscape. *Archives of Medical Research*, *54*(5), 102838. https://doi.org/10.1016/j.arcmed.2023.06.003
200. Martins, A., Londral, A., L. Nunes, I., & V. Lapão, L. (2024). Unlocking human-like conversations: Scoping review of automation techniques for personalized healthcare interventions using conversational agents. *International Journal of Medical Informatics*, *185*, 105385. https://doi.org/10.1016/j.ijmedinf.2024.105385
201. Matheson, E. L., Smith, H. G., Amaral, A. C. S., Meireles, J. F. F., Almeida, M. C., Linardon, J., Fuller-Tyszkiewicz, M., & Diedrichs, P. C. (2023). Using Chatbot Technology to Improve Brazilian Adolescents’ Body Image and Mental Health at Scale: Randomized Controlled Trial. *JMIR mHealth and uHealth*, *11*, e39934. https://doi.org/10.2196/39934
202. Maurya, R. K., Montesinos, S., Bogomaz, M., & DeDiego, A. C. (2024). Assessing the use of ChatGPT as a psychoeducational tool for mental health practice. *Counselling and Psychotherapy Research*, capr.12759. https://doi.org/10.1002/capr.12759
203. Meng, J., Rheu, M. (MJ), Zhang, Y., Dai, Y., & Peng, W. (2023). Mediated Social Support for Distress Reduction: AI Chatbots vs. Human. In *Proc. ACM Hum.-Comput. Interact.* (Vol. 7, Issue CSCW1, p. Article 72). Association for Computing Machinery.
204. Mills, R., Mangone, E., Lesh, N., Jayal, G., Mohan, D., & Baraitser, P. (2023). P015 Can chatbots improve contraceptive outcomes? A systematic review. *Posters*, A31–A31. https://doi.org/10.1136/sextrans-BASHH-2023.55
205. Mills, R., Mangone, E. R., Lesh, N., Jayal, G., Mohan, D., & Baraitser, P. (2024). Chatbots That Deliver Contraceptive Support: Systematic Review. *Journal of Medical Internet Research*, *26*, e46758. https://doi.org/10.2196/46758
206. Ming, J., Gong, D., Ngai, C. S. E., Sterling, M., Vashistha, A., & Dell, N. (2024). Wage Theft and Technology in the Home Care Context. In *Proc. ACM Hum.-Comput. Interact.* (Vol. 8, Issue CSCW1, p. Article 151). Association for Computing Machinery.
207. Misro, A., Kadoglou, N., Mishra, N., Whittington, P., & Dogan, H. (2022). *A Usability Evaluation of YouDiagnose: Artificial Intelligence Powered Physician Consultation*. https://doi.org/10.1101/2022.12.20.22283710
208. Moás, P. M., & Lopes, C. T. (2023). Automatic Quality Assessment of Wikipedia Articles—A Systematic Literature Review. In *ACM Comput. Surv.* (Vol. 56, Issue 4, p. Article 95). Association for Computing Machinery.
209. Moise, A., Centomo-Bozzo, A., Orishchak, O., Alnoury, M. K., & Daniel, S. J. (2023). Can ChatGPT Guide Parents on Tympanostomy Tube Insertion? *Children*, *10*(10), 1634. https://doi.org/10.3390/children10101634
210. Monaco, F., Vignapiano, A., Piacente, M., Pagano, C., Mancuso, C., Steardo, L., Marenna, A., Farina, F., Petrillo, G., Leo, S., Ferrara, E., Palermo, S., Martiadis, V., Solmi, M., Monteleone, A. M., Fasano, A., & Corrivetti, G. (2024). An advanced Artificial Intelligence platform for a personalised treatment of Eating Disorders. *Frontiers in Psychiatry*, *15*, 1414439. https://doi.org/10.3389/fpsyt.2024.1414439
211. Motger, Q., Franch, X., & Marco, J. (2022). Software-Based Dialogue Systems: Survey, Taxonomy, and Challenges. In *ACM Comput. Surv.* (Vol. 55, Issue 5, p. Article 91). Association for Computing Machinery.
212. Nadarzynski, T., Knights, N., Buchanan, T., Graham, C., Husbands, D., Llewellyn, C., & Ridge, D. (2022). P83 Recruiting people from racially minoritised groups into sexual health research: Lessons from the mixed-method ‘AUDITED’ study on sexual health chatbots. *Abstracts*, A61.2-A62. https://doi.org/10.1136/sextrans-BASHH-2022.128
213. Nadarzynski, T., Lunt, A., Knights, N., Bayley, J., & Llewellyn, C. (2022). O04 Perceptions and attitudes of health professionals towards the use of artificial Intelligence chatbots for sexual and reproductive health advice: A mixed-methods study. *Abstracts*, A2.1-A2. https://doi.org/10.1136/sextrans-BASHH-2022.4
214. Nadarzynski, T., Lunt, A., Knights, N., Bayley, J., & Llewellyn, C. (2023). “But can chatbots understand sex?” Attitudes towards artificial intelligence chatbots amongst sexual and reproductive health professionals: An exploratory mixed-methods study. *International Journal of STD & AIDS*, *34*(11), 809–816. https://doi.org/10.1177/09564624231180777
215. Nassar, C. M., Dunlea, R., Montero, A., Tweedt, A., & Magee, M. F. (2023). Feasibility and Preliminary Behavioral and Clinical Efficacy of a Diabetes Education Chatbot Pilot Among Adults With Type 2 Diabetes. *Journal of Diabetes Science and Technology*, 193229682311780. https://doi.org/10.1177/19322968231178020
216. Nedbal, C., Naik, N., Castellani, D., Gauhar, V., Geraghty, R., & Somani, B. K. (2024). ChatGPT in urology practice: Revolutionizing efficiency and patient care with generative artificial intelligence. *Current Opinion in Urology*, *34*(2), 98–104. https://doi.org/10.1097/MOU.0000000000001151
217. Neumann, S., Bleja, J., Krüger, T., & Grossmann, U. (2023). Participating Citizens = Smart Citizens? Applying the Human-centered Design Approach on a Digital Care Platform. In *Digit. Gov.: Res. Pract.* (Vol. 4, Issue 3, p. Article 14). Association for Computing Machinery.
218. Ng, M., Yahya, Z., Chew, G., & Issac, M. (2024). How ChatGPT can augment breast cancer care. *The Breast*, *74*, 103644. https://doi.org/10.1016/j.breast.2023.103644
219. Nguyen, M., Sedoc, J., & Taylor, C. O. (2022). Web-based Methods for Family Health History Collection. *Journal of Clinical and Translational Science*, *6*(s1), 61–61. https://doi.org/10.1017/cts.2022.189
220. Nguyen, Q. C., Aparicio, E. M., Jasczynski, M., Channell Doig, A., Yue, X., Mane, H., Srikanth, N., Gutierrez, F. X. M., Delcid, N., He, X., & Boyd-Graber, J. (2024). Rosie, a Health Education Question-and-Answer Chatbot for New Mothers: Randomized Pilot Study. *JMIR Formative Research*, *8*, e51361. https://doi.org/10.2196/51361
221. Niksirat, K. S., Korka, D., Harkous, H., Huguenin, K., & Cherubini, M. (2023). On the Potential of Mediation Chatbots for Mitigating Multiparty Privacy Conflicts—A Wizard-of-Oz Study. In *Proc. ACM Hum.-Comput. Interact.* (Vol. 7, Issue CSCW1, p. Article 142). Association for Computing Machinery.
222. Njogu, J., Jaworski, G., Oduor, C., Chea, A., Malmqvist, A., & Rothschild, C. W. (2023). Assessing acceptability and effectiveness of a pleasure-oriented sexual and reproductive health chatbot in Kenya: An exploratory mixed-methods study. *Sexual and Reproductive Health Matters*, *31*(4), 2269008. https://doi.org/10.1080/26410397.2023.2269008
223. Noble, J. M., Zamani, A., Gharaat, M., Merrick, D., Maeda, N., Lambe Foster, A., Nikolaidis, I., Goud, R., Stroulia, E., Agyapong, V. I. O., Greenshaw, A. J., Lambert, S., Gallson, D., Porter, K., Turner, D., & Zaiane, O. (2022). Developing, Implementing, and Evaluating an Artificial Intelligence–Guided Mental Health Resource Navigation Chatbot for Health Care Workers and Their Families During and Following the COVID-19 Pandemic: Protocol for a Cross-sectional Study. *JMIR Research Protocols*, *11*(7), e33717. https://doi.org/10.2196/33717
224. Noh, E., Won, J., Jo, S., Hahm, D.-H., & Lee, H. (2023). Conversational Agents for Body Weight Management: Systematic Review. *Journal of Medical Internet Research*, *25*, e42238. https://doi.org/10.2196/42238
225. Nor, A., Nur, S., Muhamad, K., Rozilawati, A., Noorazrul, Y., Muhammad, F., Nani, S., Noraini, W., Saiful, H., & Liyana, S. (2022). Radiotherapy Communication Skills Training using Chatbot-Based Prototype (SCIMORT). *Journal of Medical Imaging and Radiation Sciences*, *53*(4), S1. https://doi.org/10.1016/j.jmir.2022.10.005
226. Pal, D., Sathapornvajana, S., & Funilkul, S. (2024). Generative AI: How Well Can it Understand Conversational UX? *2024 21st International Joint Conference on Computer Science and Software Engineering (JCSSE)*, 404–411. https://doi.org/10.1109/JCSSE61278.2024.10613707
227. Pal, S., Bhattacharya, M., Lee, S.-S., & Chakraborty, C. (2024). A Domain-Specific Next-Generation Large Language Model (LLM) or ChatGPT is Required for Biomedical Engineering and Research. *Annals of Biomedical Engineering*, *52*(3), 451–454. https://doi.org/10.1007/s10439-023-03306-x
228. Palmer, C. E., Marshall, E., Millgate, E., Warren, G., Ewbank, M. P., Cooper, E., Lawes, S., Bouazzaoui, M., Smith, A., Hutchins-Joss, C., Young, J., Margoum, M., Healey, S., Marshall, L., Mehew, S., Cummins, R., Tablan, V., Catarino, A., Welchman, A. E., & Blackwell, A. D. (2024). *Combining AI and human support in mental health: A digital intervention with comparable effectiveness to human-delivered care*. https://doi.org/10.1101/2024.07.17.24310551
229. Pandey, R. R., Joshi, S. P., Nanda, B. D., Baig, K. F. M. N., Dalvi, D. S., Kelhe, Y. D., Kavedia, M., & Ingle, B. J. (2023). M-CARE: Crafting Beauty from Sanitary Waste. *2023 IEEE Engineering Informatics*, 1–5. https://doi.org/10.1109/IEEECONF58110.2023.10520512
230. Parry, M., Clarke, H., Bjørnnes, A. K., Harvey, P., Norris, C. M., Pilote, L., Price, J., Spiteri DeBonis, V., Hart, D., Nickerson, N., & O’Hara, A. (2023). Abstract 18858: At Heart *,* a Progressive Web App for Women With Heart Disease: A Pilot Randomized Controlled Trial. *Circulation*, *148*(Suppl_1). https://doi.org/10.1161/circ.148.suppl_1.18858
231. Payal, P., Dey, R., Senapati, R., Piri, J., & Mohanty, S. (2024). Medicare: A telemedicine healthcare website. *2024 7th International Conference on Circuit Power and Computing Technologies (ICCPCT)*, 1477–1482. https://doi.org/10.1109/ICCPCT61902.2024.10673273
232. Peng, R., Cao, Z., Hu, S., Liu, X., Guo, Y., Li, X., Zhang, C., & Feng, H. (2024). Frail Older Adults’ Needs and Preferences for Mobile Health Exercise Interventions Guided by Nudge Theory: AQualitative Analysis. *Journal of Clinical Nursing*, jocn.17419. https://doi.org/10.1111/jocn.17419
233. Peng, Z., Chen, Q., Shen, Z., Ma, X., & Oulasvirta, A. (2024). DesignQuizzer: A Community-Powered Conversational Agent for Learning Visual Design. In *Proc. ACM Hum.-Comput. Interact.* (Vol. 8, Issue CSCW1, p. Article 44). Association for Computing Machinery.
234. Perez-Ramos, J. G., Leon-Thomas, M., Smith, S. L., Silverman, L., Perez-Torres, C., Hall, W. C., & Iadarola, S. (2023). COVID-19 Vaccine Equity and Access: Case Study for Health Care Chatbots. *JMIR Formative Research*, *7*, e39045. https://doi.org/10.2196/39045
235. Pernencar, C., Saboia, I., & Dias, J. C. (2022). How Far Can Conversational Agents Contribute to IBD Patient Health Care—A Review of the Literature. *Frontiers in Public Health*, *10*, 862432. https://doi.org/10.3389/fpubh.2022.862432
236. Pessoa, L., Martins, L., Hsu, M., & Freitas, R. de. (2024). ZoAM GameBot: A Journey to the Lost Computational World in the Amazonia. In *J. Comput. Cult. Herit.* Association for Computing Machinery. https://doi.org/10.1145/3657303
237. Peterson, G., Moore, S., Montoya, S., Hutchison, C., Hoople, K., & Smith, C. (2023). Oncology Care at Home: A Patient-Centered Approach to Managing Care for Bone Marrow Transplant and CAR-T Cell Therapy Patients. *Blood*, *142*(Supplement 1), 3724–3724. https://doi.org/10.1182/blood-2023-190054
238. Pool, J., Indulska, M., & Sadiq, S. (2024). Large language models and generative AI in telehealth: A responsible use lens. *Journal of the American Medical Informatics Association*, *31*(9), 2125–2136. https://doi.org/10.1093/jamia/ocae035
239. Posokhov, P., Apanasovich, K., Matveeva, A., Makhnytkina, O., & Matveev, A. (2022). Personalizing Dialogue Agents for Russian: Retrieve and Refine. *2022 31st Conference of Open Innovations Association (FRUCT)*, 245–252. https://doi.org/10.23919/FRUCT54823.2022.9770895
240. Pourpanah, F., & Etemad, A. (2024). Exploring the Landscape of Ubiquitous In-home Health Monitoring: A Comprehensive Survey. In *ACM Trans. Comput. Healthcare*. Association for Computing Machinery. https://doi.org/10.1145/3670854
241. Powell, L., Nour, R., Sleibi, R., Al Suwaidi, H., & Zary, N. (2023). Democratizing the Development of Chatbots to Improve Public Health: Feasibility Study of COVID-19 Misinformation. *JMIR Human Factors*, *10*, e43120. https://doi.org/10.2196/43120
242. Priya, P., Firdaus, M., & Ekbal, A. (2024). Computational Politeness in Natural Language Processing: A Survey. In *ACM Comput. Surv.* (Vol. 56, Issue 9, p. Article 241). Association for Computing Machinery.
243. Prochaska, J. J., Vogel, E. A., Chieng, A., Baiocchi, M., Pajarito, S., Pirner, M., Darcy, A., & Robinson, A. (2023). A relational agent for treating substance use in adults: Protocol for a randomized controlled trial with a psychoeducational comparator. *Contemporary Clinical Trials*, *127*, 107125. https://doi.org/10.1016/j.cct.2023.107125
244. Purohit, R., Saineni, S., Mathai, R., Sambandan, R., Perez, R., & Bhanusali, N. (2024). AB1377 EXPLORING THE LANDSCAPE OF ARTIFICIAL INTELLIGENCE IN RHEUMATOLOGY: INSIGHTS, PERCEPTIONS, AND FUTURE CONSIDERATIONS FROM A SURVEY OF UNITED STATES RHEUMATOLOGY FELLOWS. *Scientific Abstracts*, 2041.2-2041. https://doi.org/10.1136/annrheumdis-2024-eular.976
245. Quinn, K., Leiser Ransom, S., O’Connell, C., Muramatsu, N., Marquez, D. X., & Chin, J. (2024). Assessing the Feasibility and Acceptability of Smart Speakers in Behavioral Intervention Research With Older Adults: Mixed Methods Study. *Journal of Medical Internet Research*, *26*, e54800. https://doi.org/10.2196/54800
246. Rahman, M. S., Codabux, Z., & Roy, C. K. (2024). Do Words Have Power? Understanding and Fostering Civility in Code Review Discussion. In *Proc. ACM Softw. Eng.* (Vol. 1, Issue FSE, p. Article 73). Association for Computing Machinery.
247. Rainey, J. P., Blackburn, B. E., Campbell, K. J., Anderson, L. A., & Gililland, J. M. (2024). Patient Engagement Platforms: How Technology Is Impacting Perioperative Communication and Engagement in Total Hip and Knee Arthroplasty. *Arthroplasty Today*, *27*, 101349. https://doi.org/10.1016/j.artd.2024.101349
248. Rainey, J. P., Blackburn, B. E., McCutcheon, C. L., Kenyon, C. M., Campbell, K. J., Anderson, L. A., & Gililland, J. M. (2023). A Multilingual Chatbot Can Effectively Engage Arthroplasty Patients Who Have Limited English Proficiency. *The Journal of Arthroplasty*, *38*(7), S78–S83. https://doi.org/10.1016/j.arth.2023.04.014
249. Rajesh, V., Perumal, B., Ganesh, U. S., Rajkumar, V., Kumar, D. M., & Kumar, M. (2023). Building Customer Support Chatbots With Intent Recognition. *2023 2nd International Conference on Vision Towards Emerging Trends in Communication and Networking Technologies (ViTECoN)*, 1–5. https://doi.org/10.1109/ViTECoN58111.2023.10157329
250. Ramacciotti, L. S., Cei, F., Hershenhouse, J. S., Mokhtar, D., Rodler, S., Gill, K., Strauss, D., Medina, L. G., Cai, J., Abreu, A. L., Desai, M. M., Sotelo, R., Gill, I. S., & Cacciamani, G. E. (2024). Generative AI Platform for Automating Social Media Posts From Urology Journal Articles: A Cross-Sectional Study and Randomized Assessment. *Journal of Urology*, 10.1097/JU.0000000000004199. https://doi.org/10.1097/JU.0000000000004199
251. Ranasinghe, S., De Silva, D., Mills, N., Alahakoon, D., Manic, M., Lim, Y., & Ranasinghe, W. (2024). Addressing the Productivity Paradox in Healthcare with Retrieval Augmented Generative AI Chatbots. *2024 IEEE International Conference on Industrial Technology (ICIT)*, 1–6. https://doi.org/10.1109/ICIT58233.2024.10540818
252. Ranieri, A., Di Bernardo, I., Mele, C., & Spena, T. R. (2023). Dealing with Learning Vulnerability: Service Robots to Nudge Student Engagement. *2023 IEEE International Conference on Metrology for eXtended Reality, Artificial Intelligence and Neural Engineering (MetroXRAINE)*, 1150–1155. https://doi.org/10.1109/MetroXRAINE58569.2023.10405581
253. Rapp, A., & Boldi, A. (2023). Exploring the Lived Experience of Behavior Change Technologies: Towards an Existential Model of Behavior Change for HCI. In *ACM Trans. Comput.-Hum. Interact.* (Vol. 30, Issue 6, p. Article 81). Association for Computing Machinery.
254. Rathore, B., & Chaurasia, S. (2024). Sentiment Analysis for Therapy Chatbots: A Comparison of Supervised Learning Approaches. *2024 IEEE International Conference on Contemporary Computing and Communications (InC4)*, 1–6. https://doi.org/10.1109/InC460750.2024.10649064
255. Rathore, Y., Mishra Chaturvedi, V., Sujay Madhukar, K., Karwande, V. S., Rokade, A. H., & Nagargoje, Y. (2023). Patient Engagement and Satisfaction in Ai-Enhanced Healthcare Management. *2023 International Conference on Artificial Intelligence for Innovations in Healthcare Industries (ICAIIHI)*, 1–7. https://doi.org/10.1109/ICAIIHI57871.2023.10489712
256. Ravichandran, K., & Ilango, S. K. (2023). Influence of AI Powered Gaming Developers and Analyzing Player Behavior and Enhancing User Experience. *2023 First International Conference on Advances in Electrical, Electronics and Computational Intelligence (ICAEECI)*, 1–9. https://doi.org/10.1109/ICAEECI58247.2023.10370952
257. Rawat, M., Hosseini, S. E., & Pervez, S. (2023). Sentiment Analysis for Assessing Customer Satisfaction in Chatbot Service Encounters. *2023 16th International Conference on Developments in eSystems Engineering (DeSE)*, 105–109. https://doi.org/10.1109/DeSE60595.2023.10469554
258. Richards, M., Waugh, K., Slaymaker, M., Petre, M., Woodthorpe, J., & Gooch, D. (2024). Bob or Bot: Exploring ChatGPT’s Answers to University Computer Science Assessment. In *ACM Trans. Comput. Educ.* (Vol. 24, Issue 1, p. Article 5). Association for Computing Machinery.
259. Rodriguez, D. V., Andreadis, K., Chen, J., Gonzalez, J., & Mann, D. (2024). Development of a GenAI-Powered Hypertension Management Assistant: Early Development Phases and Architectural Design. *2024 IEEE 12th International Conference on Healthcare Informatics (ICHI)*, 350–359. https://doi.org/10.1109/ICHI61247.2024.00052
260. Roffarello, A. M., & Russis, L. D. (2023). Achieving Digital Wellbeing Through Digital Self-control Tools: A Systematic Review and Meta-analysis. In *ACM Trans. Comput.-Hum. Interact.* (Vol. 30, Issue 4, p. Article 53). Association for Computing Machinery.
261. Roshani, O. V. D. E., Ayuwardhana, H. M. K. J. J., Rodrigo, P. H. M. S., Hewageegana, R. U., Fernando, H., & De Silva, D. I. (2023). Novel Approach for Enhancing Mental Well-Being Through Machine Learning Techniques. *2023 5th International Conference on Advancements in Computing (ICAC)*, 651–656. https://doi.org/10.1109/ICAC60630.2023.10417149
262. Russell, A. M., Acuff, S. F., Kelly, J. F., Allem, J., & Bergman, B. G. (2024). ChatGPT‐4: Alcohol use disorder responses. *Addiction*, add.16650. https://doi.org/10.1111/add.16650
263. Ryskaliyev, S., Tuyakbayeva, M., Kokebayeva, R., Kazakhbayeva, G., Gabitov, A., & Kuderiev, J. (2024). Managerial activity of future physical education teachers aimed at building a positive image using visual technologies. *Frontiers in Education*, *9*, 1367530. https://doi.org/10.3389/feduc.2024.1367530
264. S, H., J, S. K., D, S. D., C, S. A. A., Devi, A. S., & Velmurugan, K. J. (2023). MedLyric—Exploring Health and Well-Being Through a Website. *2023 Intelligent Computing and Control for Engineering and Business Systems (ICCEBS)*, 1–4. https://doi.org/10.1109/ICCEBS58601.2023.10448777
265. Saddik, A. E., & Ghaboura, S. (2024). The Integration of ChatGPT With the Metaverse for Medical Consultations. *IEEE Consumer Electronics Magazine*, *13*(3), 6–15. https://doi.org/10.1109/MCE.2023.3324978
266. Sağın, F. G., Özkaya, A. B., Tengiz, F., Geyik, Ö. G., & Geyik, C. (2024). Current evaluation and recommendations for the use of artificial intelligence tools in education. *Turkish Journal of Biochemistry*, *48*(6), 620–625. https://doi.org/10.1515/tjb-2023-0254
267. Sahithya, B., M S, G. P., Sahithi, B., K, M. K., Devarlla, A. C., & T R, Y. (2024). Empowering Healthcare with AI: Advancements in Medical Image Analysis, Electronic Health Records Analysis, and AI-Driven Chatbots. *2024 3rd International Conference for Innovation in Technology (INOCON)*, 1–7. https://doi.org/10.1109/INOCON60754.2024.10511753
268. Salamanca-Sanabria, A., Jabir, A. I., Lin, X., Alattas, A., Kocaballi, A. B., Lee, J., Kowatsch, T., & Tudor Car, L. (2023). Exploring the Perceptions of mHealth Interventions for the Prevention of Common Mental Disorders in University Students in Singapore: Qualitative Study. *Journal of Medical Internet Research*, *25*, e44542. https://doi.org/10.2196/44542
269. Sallam, M., Al-Farajat, A., & Egger, J. (2024). Envisioning the Future of ChatGPT in Healthcare: Insights and Recommendations from a Systematic Identification of Influential Research and a Call for Papers. *Jordan Medical Journal*, *58*(1). https://doi.org/10.35516/jmj.v58i1.2285
270. Sas, M., Denoo, M., & Mühlberg, J. T. (2023). Informing Children about Privacy: A Review and Assessment of Age-Appropriate Information Designs in Kids-Oriented F2P Video Games. In *Proc. ACM Hum.-Comput. Interact.* (Vol. 7, Issue CHI PLAY, p. Article 390). Association for Computing Machinery.
271. Schafer, M., Lachman, J. M., Gardner, F., Zinser, P., Calderon, F., Han, Q., Facciola, C., & Clements, L. (2023). Integrating intimate partner violence prevention content into a digital parenting chatbot intervention during COVID-19: Intervention development and remote data collection. *BMC Public Health*, *23*(1), 1708. https://doi.org/10.1186/s12889-023-16649-w
272. Schläpfer, S., Schneider, F., Santhanam, P., Eicher, M., Kowatsch, T., Witt, C. M., & Barth, J. (2024). Engagement With a Relaxation and Mindfulness Mobile App Among People With Cancer: Exploratory Analysis of Use Data and Self-Reports From a Randomized Controlled Trial. *JMIR Cancer*, *10*, e52386. https://doi.org/10.2196/52386
273. Schmitt, A., Wambsganss, T., & Leimeister, J. M. (2022). Conversational Agents for Information Retrieval in the Education Domain: A User-Centered Design Investigation. In *Proc. ACM Hum.-Comput. Interact.* (Vol. 6, Issue CSCW2, p. Article 486). Association for Computing Machinery.
274. Seberger, J. S., Choung, H., Snyder, J., & David, P. (2024). Better Living Through Creepy Technology? Exploring Tensions Between a Novel Class of Well-Being Apps and Affective Discomfort in App Culture. In *Proc. ACM Hum.-Comput. Interact.* (Vol. 8, Issue CSCW1, p. Article 22). Association for Computing Machinery.
275. Sedotto, R. N. M., Edwards, A. E., Dulin, P. L., & King, D. K. (2024). Engagement with mHealth Alcohol Interventions: User Perspectives on an App or Chatbot-Delivered Program to Reduce Drinking. *Healthcare*, *12*(1), 101. https://doi.org/10.3390/healthcare12010101
276. Sefidgar, Y. S., Jörke, M., Suh, J., Saha, K., Iqbal, S., Ramos, G., & Czerwinski, M. (2024). Improving Work-Nonwork Balance with Data-Driven Implementation Intention and Mental Contrasting. In *Proc. ACM Hum.-Comput. Interact.* (Vol. 8, Issue CSCW1, p. Article 74). Association for Computing Machinery.
277. Selvaskandan, H., Gee, P. O., & Seethapathy, H. (2024). Technological Innovations to Improve Patient Engagement in Nephrology. *Advances in Kidney Disease and Health*, *31*(1), 28–36. https://doi.org/10.1053/j.akdh.2023.11.001
278. Seth, I., Cox, A., Xie, Y., Bulloch, G., Hunter-Smith, D. J., Rozen, W. M., & Ross, R. J. (2023). Evaluating Chatbot Efficacy for Answering Frequently Asked Questions in Plastic Surgery: A ChatGPT Case Study Focused on Breast Augmentation. *Aesthetic Surgery Journal*, *43*(10), 1126–1135. https://doi.org/10.1093/asj/sjad140
279. Shade, M. Y., Hama, R. S., Eisenhauer, C., Khazanchi, D., & Pozehl, B. (2023). “Ask, ‘When You Do This, How Much Pain Are You In?’”: Content Preferences for a Conversational Pain Self-Management Software Application. *Journal of Gerontological Nursing*, *49*(1), 11–17. https://doi.org/10.3928/00989134-20221205-04
280. Shah, C., & Bender, E. M. (2024). Envisioning Information Access Systems: What Makes for Good Tools and a Healthy Web? In *ACM Trans. Web* (Vol. 18, Issue 3, p. Article 33). Association for Computing Machinery.
281. Sharevski, F., Loop, J. V., Jachim, P., Devine, A., & Pieroni, E. (2023). Talking Abortion (Mis)information with ChatGPT on TikTok. *2023 IEEE European Symposium on Security and Privacy Workshops (EuroS&PW)*, 594–608. https://doi.org/10.1109/EuroSPW59978.2023.00071
282. Sharma, R., Mirzakhalov, J., Bharti, P., Goyal, R., Schmidt, T., & Chellappan, S. (2023). A Friend in Need Is a Friend Indeed: Investigating the Quality of Training Data from Peers for Auto-generating Empathetic Textual Responses to Non-Sensitive Posts in a Cohort of College Students. In *ACM J. Comput. Sustain. Soc.* (Vol. 1, Issue 2, p. Article 14). Association for Computing Machinery.
283. Sharma, R., Swami, S., & Srivastava, T. (2024). PCR152 Can We Speak to GPT to Inform Patient Preference Studies? *Value in Health*, *27*(6), S324. https://doi.org/10.1016/j.jval.2024.03.2031
284. Sharma, V., Kumar, N., & Nardi, B. (2023). Post-growth Human–Computer Interaction. In *ACM Trans. Comput.-Hum. Interact.* (Vol. 31, Issue 1, p. Article 9). Association for Computing Machinery.
285. Siddiqi, D. A., Miraj, F., Raza, H., Hussain, O. A., Munir, M., Dharma, V. K., Shah, M. T., Habib, A., & Chandir, S. (2024). Development and feasibility testing of an artificially intelligent chatbot to answer immunization-related queries of caregivers in Pakistan: A mixed-methods study. *International Journal of Medical Informatics*, *181*, 105288. https://doi.org/10.1016/j.ijmedinf.2023.105288
286. Singh, A., Hrobat, M., Gui, S., Bianchi-Berthouze, N., Ley-Flores, J., Bevilacqua, F., Duran, J. R. D., Segura, E. Má., & Tajadura-JimÉnez, A. (2024). Pushed by Sound: Effects of Sound and Movement Direction on Body Perception, Experience Quality, and Exercise Support. In *ACM Trans. Comput.-Hum. Interact.* (Vol. 31, Issue 4, p. Article 53). Association for Computing Machinery.
287. Singh, A., Joshi, S., & Domb, M. (2023). Embedded Conversational AI, Chatbots, and NLP to Improve Healthcare Administration and Practices. *2023 2nd International Conference on Automation, Computing and Renewable Systems (ICACRS)*, 38–45. https://doi.org/10.1109/ICACRS58579.2023.10404985
288. Singh, A., Schooley, B., & Patel, N. (2023). Effects of User-Reported Risk Factors and Follow-Up Care Activities on Satisfaction With a COVID-19 Chatbot: Cross-Sectional Study. *JMIR mHealth and uHealth*, *11*, e43105. https://doi.org/10.2196/43105
289. Singh, M., Mittal, M., Dewan, P., Kaur, A., Kaur, G., & Gupta, A. (2024). From Text to Treatment: An Overview of Artificial Intelligence Chatbots in Healthcare. *2024 11th International Conference on Computing for Sustainable Global Development (INDIACom)*, 690–696. https://doi.org/10.23919/INDIACom61295.2024.10498497
290. Singla, A., Khanna, R., Kaur, M., Kelm, K., Zaiane, O., Rosenfelt, C. S., Bui, T. A., Rezaei, N., Nicholas, D., Reformat, M. Z., Majnemer, A., Ogourtsova, T., & Bolduc, F. (2024). Developing a Chatbot to Support Individuals With Neurodevelopmental Disorders: Tutorial. *Journal of Medical Internet Research*, *26*, e50182. https://doi.org/10.2196/50182
291. Sinha, A., Kumar, B., Kundu, R., Sharma, A., Sharma, M., & Raza, A. (2024). A Comprehensive Study of a Conversational AI-Driven Healthcare Bot with Pose Estimation for Precision Fitness Monitoring and Personalized Guidance. *2024 IEEE International Conference on Big Data &amp; Machine Learning (ICBDML)*, 1–6. https://doi.org/10.1109/ICBDML60909.2024.10577319
292. Sinha, C., Meheli, S., & Kadaba, M. (2023). Understanding Digital Mental Health Needs and Usage With an Artificial Intelligence–Led Mental Health App (Wysa) During the COVID-19 Pandemic: Retrospective Analysis. *JMIR Formative Research*, *7*, e41913. https://doi.org/10.2196/41913
293. Smith, C. E., Alam, I., Tan, C., Keegan, B. C., & Blanchard, A. L. (2022). The Impact of Governance Bots on Sense of Virtual Community: Development and Validation of the GOV-BOTs Scale. In *Proc. ACM Hum.-Comput. Interact.* (Vol. 6, Issue CSCW2, p. Article 462). Association for Computing Machinery.
294. Smith, E. D., Savage, S. K., Andrew, E. H., Martin, G. M., Kahn-Kirby, A. H., LoTempio, J., Délot, E., Cohen, A. J., Pitsava, G., Berger, S., Fusaro, V. A., & Vilain, E. (2023). *“Development and Implementation of Novel Chatbot-based Genomic Research Consent.”* https://doi.org/10.1101/2023.01.23.525221
295. Soni, H., Ivanova, J., Wilczewski, H., Bailey, A., Ong, T., Narma, A., Bunnell, B. E., & Welch, B. M. (2022). Virtual conversational agents versus online forms: Patient experience and preferences for health data collection. *Frontiers in Digital Health*, *4*, 954069. https://doi.org/10.3389/fdgth.2022.954069
296. Suffoletto, B. (2024). Deceptively Simple yet Profoundly Impactful: Text Messaging Interventions to Support Health. *Journal of Medical Internet Research*, *26*, e58726. https://doi.org/10.2196/58726
297. Sun, G. H. (2024). Prompt Engineering for Nurse Educators. *Nurse Educator*. https://doi.org/10.1097/NNE.0000000000001705
298. Sun, X., Casula, D., Navaratnam, A., Popp, A., Knopp, F., Busini, G., Wesołowski, J., Van Reeth, M., Reich, E., Wiers, R., & Bosch, J. A. (2023). Virtual Support for Real-World Movement: Using Chatbots to Overcome Barriers to Physical Activity. In P. Lukowicz, S. Mayer, J. Koch, J. Shawe-Taylor, & I. Tiddi (Eds.), *Frontiers in Artificial Intelligence and Applications*. IOS Press. https://doi.org/10.3233/FAIA230084
299. Sun, X., Teljeur, I., Li, Z., & Bosch, J. A. (2024). Can a Funny Chatbot Make a Difference? Infusing Humor into Conversational Agent for Behavioral Intervention. *ACM Conversational User Interfaces 2024*, 1–19. https://doi.org/10.1145/3640794.3665555
300. Svikhnushina, E., & Pu, P. (2022). PEACE: A Model of Key Social and Emotional Qualities of Conversational Chatbots. In *ACM Trans. Interact. Intell. Syst.* (Vol. 12, Issue 4, p. Article 32). Association for Computing Machinery.
301. Tagliaferri, L., Fionda, B., Casà, C., Cornacchione, P., Scalise, S., Chiesa, S., Marconi, E., Dinapoli, L., Di Capua, B., Chieffo, D. P. R., Marazzi, F., Frascino, V., Colloca, G. F., Valentini, V., Miccichè, F., & Gambacorta, M. A. (2024). Allies not enemies—Creating a more empathetic and uplifting patient experience through technology and art. *Strahlentherapie Und Onkologie*. https://doi.org/10.1007/s00066-024-02279-7
302. Tan, T. C., Roslan, N. E. B., Li, J. W., Zou, X., Chen, X., Ratnasari, & Santosa, A. (2023). Patient Acceptability of Symptom Screening and Patient Education Using a Chatbot for Autoimmune Inflammatory Diseases: Survey Study. *JMIR Formative Research*, *7*, e49239. https://doi.org/10.2196/49239
303. Tandon, A., Dhir, A., & Islam, N. (2024). Mobile Health Interventions for Cancer Care and Support: The Next Level of Digitalization in Healthcare? *IEEE Transactions on Engineering Management*, *71*, 6173–6189. https://doi.org/10.1109/TEM.2023.3243724
304. Tepe, M., & Emekli, E. (2024). Decoding medical jargon: The use of AI language models (ChatGPT-4, BARD, microsoft copilot) in radiology reports. *Patient Education and Counseling*, *126*, 108307. https://doi.org/10.1016/j.pec.2024.108307
305. Terblanche, N., Molyn, J., Williams, K., & Maritz, J. (2023). Performance matters: Students’ perceptions of Artificial Intelligence Coach adoption factors. *Coaching: An International Journal of Theory, Research and Practice*, *16*(1), 100–114. APA PsycInfo®. https://doi.org/10.1080/17521882.2022.2094278
306. Thakur, S. N., Sinha, A., Singh, M. K., Bagaria, M. K., Grover, R., & Shrivastava, K. (2023). Optimizing Wellness: A Comprehensive Examination of a Conversational AI-Driven Healthcare BOT for Personalized Fitness Guidance. *2023 International Conference on Artificial Intelligence for Innovations in Healthcare Industries (ICAIIHI)*, 1–8. https://doi.org/10.1109/ICAIIHI57871.2023.10489319
307. Thenmozhi, M., Rohit, K. P. R., S, A., & Chakkravarthi D, S. (2023). Chat Master Using DJANGO. *2023 IEEE Engineering Informatics*, 1–5. https://doi.org/10.1109/IEEECONF58110.2023.10520387
308. Thieme, A., Hanratty, M., Lyons, M., Palacios, J., Marques, R. F., Morrison, C., & Doherty, G. (2023). Designing Human-centered AI for Mental Health: Developing Clinically Relevant Applications for Online CBT Treatment. In *ACM Trans. Comput.-Hum. Interact.* (Vol. 30, Issue 2, p. Article 27). Association for Computing Machinery.
309. Tong, F., Lederman, R., D’Alfonso, S., Berry, K., & Bucci, S. (2022). Digital Therapeutic Alliance With Fully Automated Mental Health Smartphone Apps: A Narrative Review. *Frontiers in Psychiatry*, *13*, 819623. https://doi.org/10.3389/fpsyt.2022.819623
310. Torkamaan, H. (2023). Mood Measurement on Smartphones: Which Measure, Which Design? In *Proc. ACM Interact. Mob. Wearable Ubiquitous Technol.* (Vol. 7, Issue 1, p. Article 29). Association for Computing Machinery.
311. Totlis, T., Natsis, K., Filos, D., Ediaroglou, V., Mantzou, N., Duparc, F., & Piagkou, M. (2023). The potential role of ChatGPT and artificial intelligence in anatomy education: A conversation with ChatGPT. *Surgical and Radiologic Anatomy*, *45*(10), 1321–1329. https://doi.org/10.1007/s00276-023-03229-1
312. Tripathi, A. P., Dhaundiyal, P., Sharma, K., Sukheswala, J., Dhiman, A., & Sharma, A. K. (2024). Improving Consumer Engagement with AI Chatbots: Exploring Perceived Humanness, Social Presence, and Interactivity Factors. *2024 International Conference on Advances in Computing, Communication and Applied Informatics (ACCAI)*, 1–6. https://doi.org/10.1109/ACCAI61061.2024.10601926
313. Tsai, C.-H., Huang, C., Chen, Y.-C., Zendejas, E., Krafka, S., & Zendejas, J. (2024). Co-Design Smart Disaster Management Systems with Indigenous Communities. In *Digit. Gov.: Res. Pract.* (Vol. 5, Issue 3, p. Article 21). Association for Computing Machinery.
314. Ulrich, S., Gantenbein, A. R., Zuber, V., Von Wyl, A., Kowatsch, T., & Künzli, H. (2024). Development and Evaluation of a Smartphone-Based Chatbot Coach to Facilitate a Balanced Lifestyle in Individuals With Headaches (BalanceUP App): Randomized Controlled Trial. *Journal of Medical Internet Research*, *26*, e50132. https://doi.org/10.2196/50132
315. Ulrich, S., Lienhard, N., Künzli, H., & Kowatsch, T. (2024). A Chatbot-Delivered Stress Management Coaching for Students (MISHA App): Pilot Randomized Controlled Trial. *JMIR mHealth and uHealth*, *12*, e54945. https://doi.org/10.2196/54945
316. V, B., G, V. K., R, L., V, M., A, P., & D, K. (2024). Transforming Bite-Related Healthcare with Machine Learning, Telehealth, and Digital Integration. *2024 2nd International Conference on Artificial Intelligence and Machine Learning Applications Theme: Healthcare and Internet of Things (AIMLA)*, 1–7. https://doi.org/10.1109/AIMLA59606.2024.10531460
317. Vallurupalli, M., Shah, N. D., & Vyas, R. M. (2024). Optimizing Readability of Patient-Facing Hand Surgery Education Materials Using Chat Generative Pretrained Transformer 3.5. *The Journal of Hand Surgery*, S036350232400234X. https://doi.org/10.1016/j.jhsa.2024.05.007
318. Van Heerden, A., Bosman, S., Swendeman, D., & Comulada, W. S. (2023). Chatbots for HIV Prevention and Care: A Narrative Review. *Current HIV/AIDS Reports*, *20*(6), 481–486. https://doi.org/10.1007/s11904-023-00681-x
319. Vandelanotte, C., Trost, S., Hodgetts, D., Imam, T., Rashid, M., To, Q. G., & Maher, C. (2023). Increasing physical activity using an just-in-time adaptive digital assistant supported by machine learning: A novel approach for hyper-personalised mHealth interventions. *Journal of Biomedical Informatics*, *144*, 104435. https://doi.org/10.1016/j.jbi.2023.104435
320. Vasili, A., Schiza, E., Schizas, C. N., & Pattichis, C. S. (2024). Integrating Chatbot Functionality in a Patient Summary Based Healthcare System. In J. Mantas, A. Hasman, G. Demiris, K. Saranto, M. Marschollek, T. N. Arvanitis, I. Ognjanović, A. Benis, P. Gallos, E. Zoulias, & E. Andrikopoulou (Eds.), *Studies in Health Technology and Informatics*. IOS Press. https://doi.org/10.3233/SHTI240402
321. Victoria-Castro, A. M., Martin, M. L., Yamamoto, Y., Melchinger, H., Weinstein, J., Nguyen, A., Lee, K. A., Gerber, B., Calderon, F., Subair, L., Lee, V., Williams, A., Shaw, M., Arora, T., Garcez, A., Desai, N. R., Ahmad, T., & Wilson, F. P. (2024). Impact of Digital Health Technology on Quality of Life in Patients With Heart Failure. *JACC: Heart Failure*, *12*(2), 336–348. https://doi.org/10.1016/j.jchf.2023.09.022
322. Viduani, A., Cosenza, V., Fisher, H. L., Buchweitz, C., Piccin, J., Pereira, R., Kohrt, B. A., Mondelli, V., Van Heerden, A., Araújo, R. M., & Kieling, C. (2023). Assessing Mood With the Identifying Depression Early in Adolescence Chatbot (IDEABot): Development and Implementation Study. *JMIR Human Factors*, *10*, e44388. https://doi.org/10.2196/44388
323. Visuri, A., Van Berkel, N., & Tag, B. (2023). Wellbeing Insights in a Data-Driven Future. *2023 Fourteenth International Conference on Mobile Computing and Ubiquitous Network (ICMU)*, 1–7. https://doi.org/10.23919/ICMU58504.2023.10412212
324. Walters, N. L., Lindsey-Mills, Z. T., Brangan, A., Savage, S. K., Schmidlen, T. J., Morgan, K. M., Tricou, E. P., Betts, M. M., Jones, L. K., Sturm, A. C., & Campbell-Salome, G. (2023). Facilitating family communication of familial hypercholesterolemia genetic risk: Assessing engagement with innovative chatbot technology from the IMPACT-FH study. *PEC Innovation*, *2*, 100134. https://doi.org/10.1016/j.pecinn.2023.100134
325. Wang, H., Gupta, S., Singhal, A., Muttreja, P., Singh, S., Sharma, P., & Piterova, A. (2022). An Artificial Intelligence Chatbot for Young People’s Sexual and Reproductive Health in India (SnehAI): Instrumental Case Study. *Journal of Medical Internet Research*, *24*(1), e29969. https://doi.org/10.2196/29969
326. Wang, X., Luo, R., Liu, Y., Chen, P., Tao, Y., & He, Y. (2023). Revealing the complexity of users’ intention to adopt healthcare chatbots: A mixed-method analysis of antecedent condition configurations. *Information Processing & Management*, *60*(5), 103444. https://doi.org/10.1016/j.ipm.2023.103444
327. Wang, Y., Nagireddy, S. R., Thota, C. T., Ho, D. H., & Lee, Y. (2022). Community-in-the-loop: Creating Artificial Process Intelligence for Co-production of City Service. In *Proc. ACM Hum.-Comput. Interact.* (Vol. 6, Issue CSCW2, p. Article 285). Association for Computing Machinery.
328. Wang, Z., Shi, Y., Wang, Y., Yao, Y., Yan, K., Wang, Y., Ji, L., Xu, X., & Yu, C. (2024). G-VOILA: Gaze-Facilitated Information Querying in Daily Scenarios. In *Proc. ACM Interact. Mob. Wearable Ubiquitous Technol.* (Vol. 8, Issue 2, p. Article 78). Association for Computing Machinery.
329. Wasil, A. R., Palermo, E. H., Lorenzo-Luaces, L., & DeRubeis, R. J. (2022). Is There an App for That? A Review of Popular Apps for Depression, Anxiety, and Well-Being. *Cognitive and Behavioral Practice*, *29*(4), 883–901. https://doi.org/10.1016/j.cbpra.2021.07.001
330. Weeks, R., Cooper, L., Sangha, P., Sedoc, J., White, S., Toledo, A., Gretz, S., Lahav, D., Martin, N., Michel, A., Lee, J. H., Slonim, N., & Bar-Zeev, N. (2022). Chatbot-Delivered COVID-19 Vaccine Communication Message Preferences of Young Adults and Public Health Workers in Urban American Communities: Qualitative Study. *Journal of Medical Internet Research*, *24*(7), e38418. https://doi.org/10.2196/38418
331. Wei, J., Jiang, W., Wang, C., Yu, D., Goncalves, J., Dingler, T., & Kostakos, V. (2022). Understanding How to Administer Voice Surveys through Smart Speakers. In *Proc. ACM Hum.-Comput. Interact.* (Vol. 6, Issue CSCW2, p. Article 548). Association for Computing Machinery.
332. Wei, J., Kim, S., Jung, H., & Kim, Y.-H. (2024). Leveraging Large Language Models to Power Chatbots for Collecting User Self-Reported Data. In *Proc. ACM Hum.-Comput. Interact.* (Vol. 8, Issue CSCW1, p. Article 87). Association for Computing Machinery.
333. Wei, Y., Guo, L., Lian, C., & Chen, J. (2023). ChatGPT: Opportunities, risks and priorities for psychiatry. *Asian Journal of Psychiatry*, *90*, 103808. https://doi.org/10.1016/j.ajp.2023.103808
334. Weinberg, M., Danoff, J. R., & Scuderi, G. R. (2023). Remote Patient Monitoring Following Total Joint Arthroplasty. *Orthopedic Clinics of North America*, *54*(2), 161–168. https://doi.org/10.1016/j.ocl.2022.11.002
335. Wen, B., Norel, R., Liu, J., Stappenbeck, T., Zulkernine, F., & Chen, H. (2024). Leveraging Large Language Models for Patient Engagement: The Power of Conversational AI in Digital Health. *2024 IEEE International Conference on Digital Health (ICDH)*, 104–113. https://doi.org/10.1109/ICDH62654.2024.00027
336. Wen, K.-Y., Gomaa, S., Schnoll, M., Zhan, T., Bashir, B., & Posey, J. (2022). Pilot study of a text messaging-integrated and Chatbot-interfaced self-management program for symptom control in GI cancer patients undergoing chemotherapy. *Journal of Clinical Oncology*, *40*(16_suppl), e24117–e24117. https://doi.org/10.1200/JCO.2022.40.16_suppl.e24117
337. Wester, J., Pohl, H., Hosio, S., & Berkel, N. van. (2024). “This Chatbot Would Never...”: Perceived Moral Agency of Mental Health Chatbots. In *Proc. ACM Hum.-Comput. Interact.* (Vol. 8, Issue CSCW1, p. Article 133). Association for Computing Machinery.
338. White, B. K., Martin, A., & White, J. A. (2022). User Experience of COVID-19 Chatbots: Scoping Review. *Journal of Medical Internet Research*, *24*(12), e35903. https://doi.org/10.2196/35903
339. Whittaker, R., Dobson, R., & Garner, K. (2022). Chatbots for Smoking Cessation: Scoping Review. *Journal of Medical Internet Research*, *24*(9), e35556. https://doi.org/10.2196/35556
340. Wrightson-Hester, A.-R., Anderson, G., Dunstan, J., McEvoy, P. M., Sutton, C. J., Myers, B., Egan, S., Tai, S., Johnston-Hollitt, M., Chen, W., Gedeon, T., & Mansell, W. (2023). An artificial therapist (Manage Your Life Online) to support the mental health of youth: Co-design and case series. *JMIR Human Factors*, *10*, 22. APA PsycInfo®.
341. Wu, L., Liu, Q., Zhao, J., & Lank, E. (2023). Interactions across Displays and Space: A Study of Virtual Reality Streaming Practices on Twitch. In *Proc. ACM Hum.-Comput. Interact.* (Vol. 7, Issue ISS, p. Article 437). Association for Computing Machinery.
342. Xu, Z., Xu, H., Lu, Z., Zhao, Y., Zhu, R., Wang, Y., Dong, M., Chang, Y., Lv, Q., Dick, R. P., Yang, F., Lu, T., Gu, N., & Shang, L. (2024). Can Large Language Models Be Good Companions? An LLM-Based Eyewear System with Conversational Common Ground. In *Proc. ACM Interact. Mob. Wearable Ubiquitous Technol.* (Vol. 8, Issue 2, p. Article 87). Association for Computing Machinery.
343. Yoon, S., Goh, H., Low, X. C., Weng, J. H., & Heaukulani, C. (2024). User perceptions and utilisation of features of an AI-enabled workplace digital mental wellness platform ‘mindline at work <i>’</i>. *BMJ Health & Care Informatics*, *31*(1), e101045. https://doi.org/10.1136/bmjhci-2024-101045
344. You, Y., Tsai, C.-H., Li, Y., Ma, F., Heron, C., & Gui, X. (2023). Beyond Self-diagnosis: How a Chatbot-based Symptom Checker Should Respond. In *ACM Trans. Comput.-Hum. Interact.* (Vol. 30, Issue 4, p. Article 64). Association for Computing Machinery.
345. Yuan, F., Zhou, W., Dodge, H. H., & Zhao, X. (2023). Short: Causal structural learning of conversational engagement for socially isolated older adults. *Smart Health*, *28*, 100384. https://doi.org/10.1016/j.smhl.2023.100384
346. Zadeh, P. M., & Sattler, D. (2023). Improving Accessibility and Readability of Survey Reports in Digital Health Platforms using Conversational AI. *2023 IEEE International Conference on Bioinformatics and Biomedicine (BIBM)*, 4987–4989. https://doi.org/10.1109/BIBM58861.2023.10386018
347. Zheng, Y., Wu, Y., Feng, B., Wang, L., Kang, K., & Zhao, A. (2024). Enhancing Diabetes Self-management and Education: A Critical Analysis of ChatGPT’s Role. *Annals of Biomedical Engineering*, *52*(4), 741–744. https://doi.org/10.1007/s10439-023-03317-8
348. Zhou, H., Chen, E., Wen, S., Wang, Y., & Norel, R. (2024). Large Language Models as a Tool for Cognitive Stimulation: Chatbot Book Clubs for Seniors. *2024 IEEE International Conference on Digital Health (ICDH)*, 123–125. https://doi.org/10.1109/ICDH62654.2024.00029
