# Supplementary material for: Engaging Artificial Intelligence (AI)-based chatbots in digital health: A systematic review
Source: PLOS Digit Health. 2026 Feb 12;5(2):e0001201. doi: 10.1371/journal.pdig.0001201 (PMC12900317; doi:10.1371/journal.pdig.0001201)
Supplement: Attachment — Submitted filename: 10.22.2025 PLoS DH.docx [file pdig.0001201.s003.docx]

This study provides a systematic review and descriptive summary of studies in healthcare that have assessed the role for AI-based chatbots since the emergence of ChatGPT in 2022, inclusive of September 2024. The authors carried out this study with validated PRISMA guidelines and registered it in the PROSPERO database.

The researchers state their goals for this study with 4 research questions (“RQs”). These research questions were designed to provide a broad descriptive overview of AI-chat bots in the field of health, the methods used in the available studies, key concerns, and noted future directions.

Due to heterogeneity of studies included, the authors are unable to perform a meta-analysis however do provide relative rates of aspects of studies such as research study type, target audience. The majority of the results are descriptive and include anecdotes from the various study author conclusions.

As this is an emerging technology, having a summary of various uses of the technology, along with success and failure can help direct future research. Some of the claims made by the authors in the introduction, and in the results/discussion are not supported by the stated references. These concerns as well as other thoughts/suggestions are listed here:

*Introduction paragraph 1:*

*I am uncertain about why COVID-19 would accelerate the adoption of chatbots. With the development of large language models, there have been an increase in power for chat bots, although this development is independent of the virus. I could not find any data in Bombard et al, 2022 to support this claim.*

*Likewise, I am uncertain if the claim ‘widely used in healthcare’ can be justified. Are these widely used by providers or by patients? Are there any references to support the growth of use in this sector?*

*Table 2. Citation #7 and citation #20 are focusing on residency applications and anatomy education. Although reasonable to include these, the studies appear more geared towards academic education and administration rather than directly on digital health. It may be helpful to note in your methods that educational content was not excluded. Of course, the one limitation would be that there may be a number of chatbot-based medical school citations that were not included in your query and potential exclusion of education studies that don’t have MeSH “health” terms may have been missed.*

*Page 13: Paragraph 3. The authors state that their findings demonstrate a growing demand for transitioning from rule-based to AI-based chat bots. Their methods did not assess this claim, as they excluded any study using rule-based chat bots so it is unclear if demand is growing or receding. This paragraph could be better stated as just describing the two themes that the authors proposed are shown in the available studies (i.e. omit the final sentence and remove terms such as trends or growing demand).*

*Page 14: Paragraph 3. The authors state that “patients and everyday individual have adopted AI-based chatbots for various purposes”. The research studies cited do not all describe patient adaptation. For example, Lim et al (2024) had investigators enter prompts into commercially available large language models and authors analyzed the output, so this was not a study of patient adaptation. In addition Gordon et al. (2024) did a similar study where fictitious applications were developed by investigator and admissions faculty were surveyed. This did not show that this was being adopted into clinical practice. Patient adopting the technology would need to be demonstrated by patients using a technology spontaneously or sharing interest in a survey-based study, measured by looking at patient use, satisfaction, plan for future use etc.*

*Page 19: Paragraph 1. One of the major cited concerns with using large language model AI chat bots is the risk of “hallucination” (fabrication of data or answers to address the prompt when an obvious answer cannot be cited). In the final you state that most of the quantified studies found the text quality accurate and evidence-based. Are you able to summarize the quantified findings on the degree of accuracy. Are you able to quantify how often these studies noted hallucinations? If absolute numbers are hard to elucidate, at least some statement on the studies’ noting of hallucinations should be addressed.*

*Page 22: Paragraph 2. The authors state that “larger randomized controlled trials in more diverse cultural and demographic settings are needed”. To address which questions in particular? As their systematic review included studies from the earliest incorporation Chat GPT (page 5), one would expect most of the studies to be small at this time. Assessment of safety with smaller studies is appropriate for an emerging technology. Although I do not disagree that larger studies with broader demographics would increase our understanding, I would suggest rephrasing this statement to acknowledge the nascent nature of the technology and priority on ensuring accuracy and safety.*

Due to the very recent emergence of Chat GPT, and the number of restrictions with protected patient information being entered into an algorithm that by nature learns and retains all inputs, initial studies using commercial large language models would not contain protected patient health information. This should be acknowledged by the authors as well as whether or not there are emerging large language models that protect patient privacy that overcome this barrier to research. Additional limitations beyond just the small number of studies should be acknowledged by the authors, including limitations of study selection, short time frame (2022-2024), as well as rapid development of the Chat GPT models (capabilities of these tools are vastly improved today compared to their initial introduction).

I would like to commend the authors for the undertaking and attempt to find a signal among noise in the emerging studies with AI chat bots. I would recommend ensuring citations support statements in the body of the text and clarity on whether education/administration was a focus on their study in addition to addressing the other queries above.
