# Supplementary material for: Engaging Artificial Intelligence (AI)-based chatbots in digital health: A systematic review
Source: PLOS Digit Health. 2026 Feb 12;5(2):e0001201. doi: 10.1371/journal.pdig.0001201 (PMC12900317; doi:10.1371/journal.pdig.0001201)
Supplement: Attachment — Submitted filename: 1-Revision table.docx [file pdig.0001201.s005.docx]

| **Reviewers' Comments:** | **Responses** |
| --- | --- |
| Reviewer #1: |  |
| 1. The abstract should more clearly state the number of articles contained in the total number collected (i.e., "A total of 348 articles were collected, 20 of which were analysed.") to facilitate immediate comprehension. | We have added the following to the abstract: “A total of 348 articles were collected from eight databases—PubMed/MEDLINE, EMBASE, PsycINFO, CINAHL, IEEE, the ACM Digital Library, Scopus, and Web of Science—of which 20 were analysed.” Please see Page 1. |
| 2. The article refers to the limited theoretical framework discussion but fails to detail well enough why that constitutes a limitation or make concrete suggestions regarding which frameworks (beyond the couple of them they did mention) would be most beneficial for future engagement and efficacy studies to utilize. | Thank you for this helpful suggestion. We acknowledge that the discussion of theoretical frameworks was insufficiently developed in the selected literature, which is one of our key findings after reviewing the field. In the revised manuscript, we have elaborated on why limited engagement with theory represents a limitation for the field and proposed relevant frameworks. Please refer to Page 24. |
| 3. While the paper categorizes AI models (Table 4) and mentions LLMs like GPT-3.5 and GPT-4, the separation of "AI-based chatbots specifically for healthcare" (Group 1) from "AI models of ChatGPT" (Group 2) and "Compared different AI models" (Group 3) could be clearer, especially when discussing technical foundations (e.g., the exact position of LLMs over trained data libraries in Group 1). | We agree and have added the exact position of LLMs in relation to trained data libraries in Group 1 for greater clarity. Please refer to Table 5 on Pages 11-12. |
| 4. The article correctly lists chatbot safety as a topmost area but also suggests that it only covers 5% of the articles covered, and there's a vast research gap around the same. This underrepresentation warrants more emphasis in the Discussion as an area of high priority, urgent investigation. The issue of a lack of wider applicability and generalisability (RQ4) is a reiterative topic but seems to be slightly repetitive across the limitations and future directions. | We have expanded the discussion, emphasising that this underrepresentation warrants greater attention in the revised manuscript as an area of high priority and urgent investigation. Please see Pages 24-25.  In addition, we would clarify that the issue of limited wider applicability and generalisability emerged as a research concern from our review of the selected literature, and we therefore propose it as part of the future research agenda outlined in this article. |
| 5. The usage of terms such as "AI-based chatbots" and "conversational agents" should be standardized. As defined, sometimes the difference is not clear-cut. For example, in the Introduction, it would be helpful to clearly declare that the emphasis will be on the AI-based subset once the two categories are introduced. | We have reviewed the entire manuscript to ensure that this work consistently uses the term 'AI-based chatbot'. We have also added the sentence, “This current work will focus on the AI-based subset,” to the Introduction. Please refer to Page 2. |
| 6. It directly addresses issues of data security, privacy, and open data sharing in healthcare, one of the significant ethical issues raised in the systematic review. It presents an advanced technical solution (Blockchain + MOA) for issues present in current AI models. It is suggested to refer from the paper: “Blockchain-Enabled Healthcare Optimization: Enhancing Security and Decision-Making Using the Mother Optimization Algorithm”. | We have cited this article in the revised discussion. Please refer to Pages 24-25. |
| 7. Integrate Recent AI Technical Developments as the article mentions potential future research directions, such as the creation of LLMs designed for a particular clinical area. This section (RQ4) would be greatly enhanced by including technical concepts behind such advancements, e.g., utilizing specialized AI algorithms or blockchain in secure data processing, which is relevant directly to the "safety" and "data security" concerns identified. Action: Include a reference to an AI or machine learning approach which addresses the data security, efficiency, or computational complexity problems of AI in healthcare. | The article *“Blockchain-Enabled Healthcare Optimization: Enhancing Security and Decision-Making Using the Mother Optimization Algorithm”* has been cited in the revised discussion. Please refer to Pages 24-25. |
| Reviewer #2: |  |
| Introduction paragraph 1: I am uncertain about why COVID-19 would accelerate the adoption of chatbots. With the development of large language models, there have been an increase in power for chat bots, although this development is independent of the virus. I could not find any data in Bombard et al, 2022 to support this claim.  Likewise, I am uncertain if the claim ‘widely used in healthcare’ can be justified. Are these widely used by providers or by patients? Are there any references to support the growth of use in this sector? | For greater rigour, we have removed such phrases in the revised manuscript. Please see Page 2. |
| Table 2. Citation #7 and citation #20 are focusing on residency applications and anatomy education. Although reasonable to include these, the studies appear more geared towards academic education and administration rather than directly on digital health. It may be helpful to note in your methods that educational content was not excluded. Of course, the one limitation would be that there may be a number of chatbot-based medical school citations that were not included in your query and potential exclusion of education studies that don’t have MeSH “health” terms may have been missed. | We clarified that medical education and administration fall within the scope of digital health, as using digital tools in medical education and administration. In the methods, we added: “Digital health communication is central to contemporary healthcare, supporting the dissemination of public health messages, the delivery of health education, the exchange of health data between patients and providers, and the overall streamlining of healthcare services (Maher & Singh, 2024). Given the interdisciplinary nature of digital health communication (Maher & Singh, 2024), we combined databases from health and medicine, electronics engineering and computing research, as well as general databases to ensure a comprehensive coverage of the literature.” Please see this revision on Page 4.  For the limitations pointed out from the reviewers, we added “The potential exclusion of studies that don’t have MeSH “health” terms may have been missed from PubMed.” Please refer to the Page 24. |
| Page 13: Paragraph 3. The authors state that their findings demonstrate a growing demand for transitioning from rule-based to AI-based chat bots. Their methods did not assess this claim, as they excluded any study using rule-based chatbots, so it is unclear if demand is growing or receding. This paragraph could be better stated as just describing the two themes that the authors proposed are shown in the available studies (i.e. omit the final sentence and remove terms such as trends or growing demand). | We agree and have therefore omitted the final sentence and removed terminology such as “trends” and “growing demand. Please refer to the Page 13. |
| Page 14: Paragraph 3. The authors state that “patients and everyday individual have adopted AI-based chatbots for various purposes”. The research studies cited do not all describe patient adaptation. For example, Lim et al (2024) had investigators enter prompts into commercially available large language models and authors analysed the output, so this was not a study of patient adaptation. In addition, Gordon et al. (2024) did a similar study where fictitious applications were developed by investigator and admissions faculty were surveyed. This did not show that this was being adopted into clinical practice. Patient adopting the technology would need to be demonstrated by patients using a technology spontaneously or sharing interest in a survey-based study, measured by looking at patient use, satisfaction, plan for future use etc. | We appreciate the reviewer pointing this out and have rewritten these sentences for accuracy in the revised section. Please see Page 14. |
| Page 19: Paragraph 1. One of the major cited concerns with using large language model AI chat bots is the risk of “hallucination” (fabrication of data or answers to address the prompt when an obvious answer cannot be cited). In the final you state that most of the quantified studies found the text quality accurate and evidence-based. Are you able to summarize the quantified findings on the degree of accuracy. Are you able to quantify how often these studies noted hallucinations? If absolute numbers are hard to elucidate, at least some statement on the studies’ noting of hallucinations should be addressed. | We agree with this suggestion and have incorporated a summary of quantified findings to improve the rigour of the revised manuscript. Please refer to Page 19. |
| Page 22: Paragraph 2. The authors state that “larger randomized controlled trials in more diverse cultural and demographic settings are needed”. To address which questions in particular? As their systematic review included studies from the earliest incorporation Chat GPT (page 5), one would expect most of the studies to be small at this time. Assessment of safety with smaller studies is appropriate for an emerging technology. Although I do not disagree that larger studies with broader demographics would increase our understanding, I would suggest rephrasing this statement to acknowledge the nascent nature of the technology and priority on ensuring accuracy and safety. | Including only small, homogeneous user groups from specific geographic locations limited the broader applicability and generalisability of AI-based chatbots. For clarity, we have rephrased this as: ‘These lack broader applicability and generalisability; although they provide early-stage insights from the technology development perspective, we suggest that randomised controlled trials in more diverse cultural and demographic settings are needed to determine whether they are truly effective for a culturally diverse range of users.’ Please see Page 22.  For discussing safety concerns, please refer to Page 21, as well as the subsection titled “Calls for Research into the Ethics, Safety, and Regulation of AI Language Models” on Page 23. |
| Due to the very recent emergence of Chat GPT, and the number of restrictions with protected patient information being entered into an algorithm that by nature learns and retains all inputs, initial studies using commercial large language models would not contain protected patient health information. This should be acknowledged by the authors as well as whether or not there are emerging large language models that protect patient privacy that overcome this barrier to research. Additional limitations beyond just the small number of studies should be acknowledged by the authors, including limitations of study selection, short time frame (2022-2024), as well as rapid development of the Chat GPT models (capabilities of these tools are vastly improved today compared to their initial introduction). | We appreciate this point and have included these in the discussion to state this limitation of current manuscripts. Please refer to Pages 24-25. |
| I would like to commend the authors for the undertaking and attempt to find a signal among noise in the emerging studies with AI chat bots. I would recommend ensuring citations support statements in the body of the text and clarity on whether education/administration was a focus on their study in addition to addressing the other queries above. | We clarified that medical education and administration fall within the scope of digital health. Please refer to the response above for the remaining queries. |
| Reviewer #3: |  |
| A key contextual limitation is that the work appears to have been undertaken around the same time as the systematic review by Bedi et al., published in JAMA (“Testing and Evaluation of Healthcare Applications of Large Language Models”). While the JAMA review is broader in scope, it employs a clearly rationalised search strategy and presents a framework for classifying and synthesising LLM research that is highly relevant here. The present paper would benefit from explicitly acknowledging and referencing that work and ideally aligning aspects of its analysis and synthesis with it. In the present manuscript, Feng and colleagues identify four critical domains being evaluated by AI researchers, whereas Bedi et al. highlight several additional important concepts. The authors need to clarify how their review provides distinct or deeper insights within the narrower domain of AI-based health chatbots. At times, the paper appears less detailed in areas where greater specificity could add value – this is not to say in other areas they don’t provide more detail. | We have cited this work in the revised discussion section to clarify how our review provides distinct or deeper insights within the narrower domain of AI-based health chatbots. Please refer to Pages 24-25. |
| Some inconsistency in the terminology and framing of “AI-based chatbots” warrants attention throughout—particularly the implicit suggestion that the tools captured in the literature represent the only available health chatbots. For instance, our recent Frontiers in Public Health (https://www.frontiersin.org/journals/public-health/articles/10.3389/fpubh.2025.1584348/full) paper identified more than a thousand publicly accessible, health-focused GPTs, highlighting that many such chatbots exist outside the peer-reviewed literature. While these may fall beyond the inclusion criteria of this systematic review, their existence should be acknowledged as contextual evidence of the broader, evolving chatbot ecosystem. | We appreciate this point and have cited this work in the discussion to state this limitation of current manuscripts. Please refer to Pages 24-25. |
| The supplementary material could be improved to better share the search process and ensure that all data underlying the findings in their manuscript are fully accessible. | We have included supplementary material to enhance the data shareability of this study. |
| The English is generally clear, but there are some difficulties in following the specified aims and layout of the results (and methods), which affects readability from my perspective. | We have proofread these sections in the revised one. |
| Finally, although the paper identifies four main research areas—text quality, clinical efficacy, user engagement, and safety—the treatment of “safety” appears largely focused on response accuracy, with limited consideration of the broader safety implications of chatbot use. This is chiefly because such papers, such as the above two, were not part of the literature captured by the review conducted herein. | We have discussed this issue for limited consideration of the broader safety implications of chatbot use in the updated discussion. Please refer to Pages 24-25. |
